# Acrylamide-forming potential of potatoes grown at different locations, and the ratio of free asparagine to reducing sugars at which free asparagine becomes a limiting factor for acrylamide formation

**DOI:** 10.1016/j.foodchem.2016.09.199

**Published:** 2017-04-01

**Authors:** Nira Muttucumaru, Stephen J. Powers, J. Stephen Elmore, Andrew Dodson, Adrian Briddon, Donald S. Mottram, Nigel G. Halford

**Affiliations:** a Plant Biology and Crop Science Department, Rothamsted Research, Harpenden, Hertfordshire AL5 2JQ, United Kingdom; b Computational and Systems Biology Department, Rothamsted Research, Harpenden, Hertfordshire AL5 2JQ, United Kingdom; c Department of Food and Nutritional Sciences, University of Reading, Whiteknights, Reading RG6 6AP, United Kingdom; d AHDB Potatoes, Sutton Bridge Crop Storage Research, East Bank, Sutton Bridge, Spalding, Lincolnshire PE12 9YD, United Kingdom

**Keywords:** Acrylamide, Asparagine, crop composition, Food safety, Post-harvest storage, Processing contaminant, Reducing sugars

## Abstract

Acrylamide is produced from free asparagine and reducing sugars during high-temperature cooking and food processing, and potato products are major contributors to dietary acrylamide intake. The present study analysed twenty varieties of potatoes grown at two sites (Doncaster and Woburn) in the United Kingdom to assess the effect of location of cultivation on acrylamide-forming potential. Analysis of variance revealed a full site by variety nested within type (French fry, boiling and crisping) by storage interaction for acrylamide (p < 0.003, F-test), reducing sugars and total sugars (p < 0.001, F-test). There was much greater free asparagine in potatoes grown at the Doncaster site compared with the Woburn site. Modelling of the relationship between the ratio of free asparagine to reducing sugars and the levels of acrylamide identified a value of 2.257 ± 0.149 as the tipping point in the ratio below which free asparagine concentration could affect acrylamide formation.

## Highlights

• Location of cultivation affects potato composition and acrylamide-forming potential.
• Effects of variety and storage interact with those of location.
• Dramatic differences in free asparagine concentration in potatoes grown at two sites.
• Concentration of reducing sugars is the primary determinant of acrylamide formation.
• Ratio of free asparagine to reducing sugars determines whether free asparagine affects acrylamide formation.

## 1 Introduction

Acrylamide (C_3_H_5_NO) is a processing contaminant that is produced in the Maillard reaction, a series of non-enzymic reactions between reducing sugars such as glucose and fructose, and free amino acids (Halford et al., 2011, Mottram, 2007, Nursten, 2005). The reaction occurs at the high temperatures generated by frying, baking, roasting or high-temperature processing, and is also responsible for the production of melanoidin pigments and complex mixtures of compounds that impart the flavours and aromas that are associated with fried, baked and roasted foods. Acrylamide forms principally *via* the deamination and decarboxylation of asparagine (Mottram et al., 2002, Stadler et al., 2002, Zyzak et al., 2003): free asparagine and reducing sugars can therefore be regarded as its precursors (in fact the carbon skeleton is derived entirely from asparagine). In potato, the relationship between precursor concentration and acrylamide formation is complex, with reducing sugars being the major determinants of acrylamide-forming potential in most datasets but free asparagine contributing to the variance in some (Amrein et al., 2003, Becalski et al., 2004, De Wilde et al., 2005, Elmore et al., 2007, Elmore et al., 2010, Halford et al., 2012b, Muttucumaru et al., 2013, Muttucumaru et al., 2014b, Shepherd et al., 2010). Notably, two studies have shown free asparagine concentration to correlate significantly with acrylamide-forming potential in French fry but not crisping (US chipping) varieties, probably because French fry varieties contain higher concentrations of sugars (Halford et al., 2012b, Muttucumaru et al., 2014b).

Understanding the relationship between precursor concentration and acrylamide formation is important because the presence of acrylamide in popular foods is now one of the most pressing problems facing the food industry. Acrylamide is classified as a Group 2A, ‘probably carcinogenic to humans’ carcinogen and the latest report from the European Food Safety Authority (EFSA)’s Expert Panel on Contaminants in the Food Chain (CONTAM) stated that the margins of exposure for acrylamide indicate a concern for neoplastic effects based on animal evidence (EFSA Panel on Contaminants in the Food Chain (CONTAM), 2015). The European Commission issued ‘indicative’ levels for the presence of acrylamide in food in 2011 and revised them downwards for many products in 2013 (European Commission, 2013). Currently the indicative levels for potato crisps and French fries are 1000 and 600 μg per kg (parts per billion), respectively. Indicative levels are not regulatory limits or safety thresholds, although they have sometimes been misinterpreted as such by journalists, resulting in damaging publicity for companies whose products have been found to exceed the indicative level. Furthermore, at the time of writing the Commission is considering its options for strengthening its risk management measures in response to the CONTAM report. In the USA, the Food and Drug Administration has not imposed restrictions but has developed an ‘action plan’ with a number of goals, including identifying means to reduce exposure, and has issued a ‘guidance’ document for industry (FDA, 2016).

Potato, coffee and cereal products are the major contributors to dietary acrylamide intake. In Europe, potato products account for between 18.3% of intake for adults in France and 67.1% for adults in the UK (European Food Safety Authority, 2011), the difference between the two countries being attributable to contrasting dietary preferences. Between 60 and 80% of this intake is from French fries, with crisps and oven-cooked potatoes accounting for the rest.

The food industry has devised many strategies for reducing acrylamide formation by modifying food processing and these have been compiled in a ‘Toolbox’ produced by Food Drink Europe (2013). Analysis of manufacturers’ data on acrylamide levels in potato crisps in Europe showed a clear, statistically significant downward trend for mean levels of acrylamide from 763 (±91.1) μg per kg in 2002 to 358 (±2.5) μg per kg in 2011, a decrease of 53% (Powers, Mottram, Curtis, & Halford, 2013). This was taken as evidence of the effectiveness of the ‘Toolbox’. However, the effect of seasonality arising from the influence of potato storage on acrylamide levels was evident in the study, with acrylamide in the first six months of the year being, on average, 160 μg per kg higher than in the second six months (Powers et al., 2013). This was consistent with the results of studies showing a significant effect of storage on reducing sugar concentration and acrylamide-forming potential (De Wilde et al., 2005, Halford et al., 2012b, Muttucumaru et al., 2014b) and with the advice that potatoes should only be used for crisping, frying and roasting within their recommended storage window (Halford et al., 2012b). It also highlighted the challenge faced by the food industry in processing such a variable raw material to give a level of acrylamide in the end product that consistently complies with indicative levels.

Enabling potatoes to be produced with lower and more predictable acrylamide-forming potential is now a target for potato breeders and agronomists, because doing so would help the food industry to comply with indicative levels or regulatory limits, should they be introduced, without costly changes to manufacturing processes. This requires the identification or breeding of varieties that stay consistently low in acrylamide-forming potential through a range of environments and conditions, and the development of best crop management practice (Halford et al., 2012a). Both nutrition and water availability affect the acrylamide-forming potential of potatoes (De Wilde et al., 2006, Muttucumaru et al., 2015, Muttucumaru et al., 2013), with increased nitrogen and irrigation generally leading to more acrylamide-forming potential, although there are differences in the ways that the three types of potato (boiling, crisping and French-fry) respond and in the responses of varieties within each type.

In the present paper we report the effect of location on acrylamide-forming potential by comparing the levels of acrylamide in tuber flour prepared from twenty varieties of field-grown potatoes grown in 2011 at a site near Doncaster in the North of England, UK, with previously-published data on a replica field trial grown in the same year at Woburn in the south of England (Elmore et al., 2015). The study investigates the interacting effects of location, variety and storage on acrylamide-forming potential. Furthermore, the contribution of the different precursors to acrylamide formation in potatoes from the two sites are considered, enabling the identification of a tipping point in the ratio of free asparagine to reducing sugars, above which reducing sugar concentration is the main determining factor but below which free asparagine concentration contributes to the variance in acrylamide-forming potential.

## 2 Materials and methods

### 2.1 Potatoes

Twenty potato (*Solanum tuberosum*) varieties were grown in a field trial at Thorn Bank, Doncaster, North Lincolnshire, UK (Grid reference: SE723015; 53°30′20″N, 0°54′40′W; soil type: peaty sand) in 2011. The field trial was a replica of a simultaneous trial at Woburn in Bedfordshire, UK (Grid reference SP968364; 52°01′06″N, 0°35′30′W; soil type: sandy clay loam) (Elmore et al., 2015). For both trials a randomised block design was used with three blocks. Hence, three plots of each variety were grown, with each plot serving as a replicate. Planting took place in April 2011 and the tubers were harvested in September and October 2011, according to whether they were early-, mid- or late-maturing varieties and to when canopy senescence was complete. Fertiliser was applied at planting at a rate of 200 kg ha^−1^ nitrogen, 100 kg ha^−1^ phosphorus, 300 kg ha^−1^ potassium and 100 kg ha^−1^ sulphur, and the field was irrigated.

The potatoes were stored at 8 °C for either two or six months at the Agriculture and Horticulture Development Board Sutton Bridge Crop Storage Research facility. They were treated with the anti-sprout agent chlorpropham, with applications just after storage commenced and two further applications for the six-month samples. All applications were made at a rate of 28 mL per tonne, using ProLong (50% w/v chlorpropham in methanol; UPL Europe Ltd., Warrington, UK).

### 2.2 Free amino acids, sugars and acrylamide formation

Free amino acids and sugars were measured in flour samples prepared from individual freeze-dried tubers using the methods described by Halford et al. (2012b) and Elmore et al. (2015). Free amino acids were derivatised using the EZ-Faast system (Phenomenex, Torrance, CA, USA) and analysed by GC–MS. Note that it is not possible to measure arginine by this method. Sugars were extracted and quantified by ion exchange chromatography with pulsed amperometric detection.

Acrylamide concentration was measured in tuber flour heated for 20 min at 160 °C (Halford et al., 2012b). The heated flour (∼0.5 g, accurately weighed) was extracted with water (40 mL, containing 50 μg/L ^13^C_3_-acrylamide internal standard) at room temperature in a 50 mL centrifuge tube. After shaking for 20 min, tube and contents were centrifuged for 15 min at 15 °C and 9072 rcf. Aqueous extract was removed and 2 mL was passed through a 0.2 μm syringe filter into a 2 mL vial. Samples were analysed by liquid chromatography–mass spectrometry/mass spectrometry (LC–MS/MS) using an Agilent 1200 high-performance liquid chromatography (HPLC) system with 6410 triple quadrupole mass spectrometer with electrospray ion source in positive ion mode as described by Halford et al. (2012b).

### 2.3 Statistical analyses

The data for analysis comprised concentrations of free amino acids (mmol kg^−1^), acrylamide (μg/kg) and sugars (glucose, fructose and sucrose) (mmol kg^−1^) for all samples. The total sugar and total reducing sugar (glucose and fructose) content were also calculated, as well as the total amino acid content and the ratio of free asparagine to the total free amino acid content. The data from the Doncaster field trial and the concurrent one at the site in Woburn (Elmore et al., 2015) were analysed together to assess the effects of site interacting with those of type (crisping, French fry and boiling), variety within type, and storage. There were therefore 240 observations for each variable that was measured, comprising a factorial treatment structure with two sites by 20 varieties by two storage conditions with three replicates.

Analysis of variance (ANOVA) was applied to the data, taking account of the randomised block design structure at the two sites and testing (F-tests) the main effects and interactions between the factors of site, type, variety nested in type, and storage condition. The relevant tables of means were drawn up with standard errors of the difference (SED) values on the corresponding degrees of freedom (df), thus allowing comparison of particular pairs of means of interest by using the least significant difference (LSD) value at the 5% (p = 0.05) level of significance. A natural log transformation was used to account for some observed heterogeneity of variance in the raw data across the treatment combinations for all responses except sucrose. For the amino acids a small adjustment (0.005) was used to account for zero observations under log transformation.

The log transformation was required to ensure that the data pertained to a Normal distribution with broadly constant variance over the treatment combinations and with additivity of effects between treatment combinations. These are the three assumptions made for application of ANOVA. As a common transformation was applied to all observations, the comparative values between treatments were not altered and comparisons between them remained valid (Gomez & Gomez, 1984). Back-transformed means were then calculated to consider the differences between treatments on the raw scale.

Pearson’s correlations (*r*) were calculated between pairs of measured variables and these were tested using the F-test. This considered the strength of associations and in particular those between precursors and acrylamide. Correlations were considered for all data together and also for the 2-month and 6-month storage data separately.

Subsequently, the relationship between acrylamide and the ratio of free asparagine to reducing sugars was investigated using a broken-stick regression model fitted using ordinary least squares, with the requirement of separate estimates of model parameters for the effects of site and storage being tested *via* F-tests. This type of regression modelling has been used successfully in various biological applications; for example, to model the numbers of leaves on oilseed rape plants (Powers, Pirie, Latunde-Dada, & Fitt, 2010). Residuals from the best model that was derived were checked for conformation to a Normal distribution and consistency of variance with increasing fitted values.

The GenStat (17th edition, (c) VSN International Ltd, Hemel Hempstead, UK) statistical package was used for the analysis.

## 3 Results and discussion

### 3.1 Free amino acid and sugar concentrations in potatoes grown at two different sites in the UK

Twenty varieties of potatoes were grown at a site near Doncaster, in the north of England, UK, in 2011, in a replica of a field trial at Woburn, Bedfordshire, southern England, UK, some results for which have already been published (Elmore et al., 2015). The Doncaster site is approximately 120 miles almost directly north of the Woburn site. The two sites also differ in soil type, with peaty sand at the Doncaster site and sandy loam at the Woburn site. The potatoes were harvested in September and October of 2011, kept in storage at 8 °C in a commercial potato store, and analysed after two and six months.

The varieties included in the study were Lady Claire, Lady Blanca, Lady Olympia, Lady Rosetta, Daisy, King Edward, Maris Piper, Fontane, Hermes, Markies, Harmony, Pentland Dell, Desiree, Challenger, Ramos, Innovator, Umatilla Russet, Russet Burbank, Saturna and Verdi. Verdi, Lady Olympia, Lady Blanca and Umatilla Russet have been introduced to the UK market recently but the others are well-established and account for about half of the total UK potato production (Elmore et al., 2015).

Hermes, Lady Claire, Lady Rosetta, Saturna and Verdi are crisping varieties, while Challenger, Daisy, Desiree, Fontane, Innovator, King Edward, Lady Blanca, Lady Olympia, Maris Piper, Pentland Dell, Ramos, Russet Burbank and Umatilla Russet are French fry varieties. Markies is predominantly a French fry variety but recently has established a market for crisping because of its stability during storage (Halford et al., 2012b). Harmony is suitable only for boiling because of its high sugar content (Muttucumaru et al., 2014b).

The potatoes were analysed for free amino acid and sugar (glucose, fructose and sucrose) concentration, and the amount of acrylamide formed was measured in flour after heating for 20 min at 160 °C. Acrylamide levels in crisps prepared from the Woburn potatoes have been reported previously (Elmore et al., 2015) but not acrylamide levels in heated flour. Acrylamide formation in crisps is directly comparable to ‘real-world’ food processing, but the flour method gives high levels of acrylamide formation and provides a good, consistent indication of acrylamide-forming potential in different raw materials (Halford et al., 2012b). The full dataset for the Doncaster site and the flour acrylamide data for the Woburn site are given in Supplementary File S1. Analysis of variance (ANOVA) was applied to the data from the two sites, and the p-values for the main effects and interactions between the terms of site (S), type (T), variety (V) and storage (St) tested in the ANOVA are given in Table 1, while the full set of relevant means tables from the analysis is given in Supplementary File S2.

**Fig. 1:**
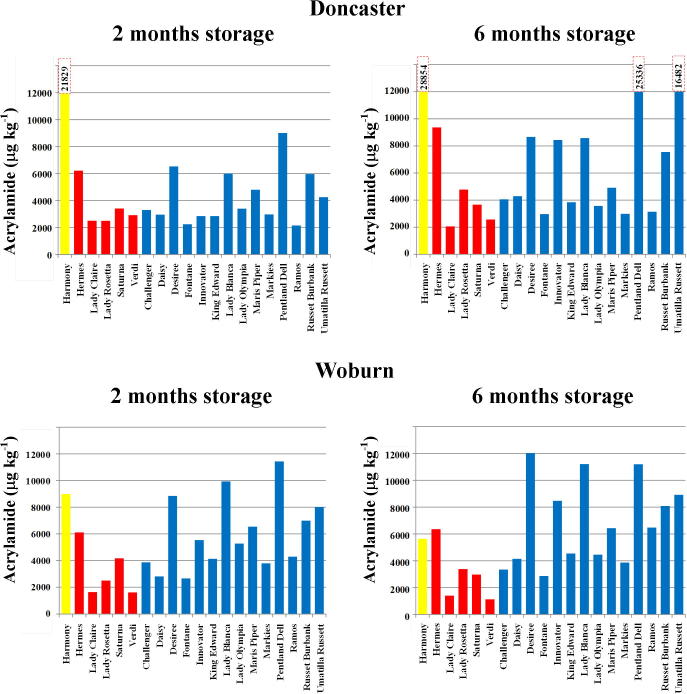
Acrylamide formation (μg kg^−1^ dry weight) (back-transformed means from analysis of variance) in tuber flour heated to 160 °C for 20 min for twenty potato varieties grown at sites near Doncaster and Woburn, UK, and stored at 8 °C for two or six months, as indicated. The boiling type (Harmony) is shown in yellow, crisping type in red and French fry type in blue. The columns for Harmony in the top panels and for Pentland Dell and Umatilla Russet in the top right panel are truncated to allow them to be shown on the same scale as the others, and the actual figures are given above each column. For statistical analysis see Table 1, Table 2. (For interpretation of the references to colour in this figure legend, the reader is referred to the web version of this article.)

There was a full site by variety (nested within type) by storage interaction for acrylamide (p = 0.003, F-test), glucose (p = 0.001, F-test), fructose (p < 0.001, F-test), reducing sugars (p < 0.001, F-test), and total sugars (p < 0.001, F-test) (Table 1). The means for acrylamide, glucose, fructose and total reducing sugars on the natural log scale and their back-transformed values are given in Table 2, while the means for total sugars are given in Table S1. The mean acrylamide concentrations are shown graphically in Fig. 1: acrylamide increased with storage for all varieties except for Lady Claire and Verdi at both sites, and for Harmony, Saturna, Challenger, Lady Olympia, Maris Piper and Pentland Dell from the Woburn site. Of these, the reductions for Harmony and Verdi from the Woburn site were significant (p < 0.05, LSD). The greatest increase in acrylamide after storage was for Umatilla Russet from the Doncaster site and for Innovator at the Woburn site (see values in bold italics in Table 2).

This storage effect is consistent with several previous studies (De Wilde et al., 2005, Halford et al., 2012b, Muttucumaru et al., 2014b), which have all attributed the rise in acrylamide-forming potential during storage to the increase in reducing sugar concentration brought about by cold sweetening (Sowokinos, 1990). The effect was not observed by Elmore et al. (2015) for the Woburn potatoes. However, inclusion of both the Woburn and Doncaster datasets in this analysis could explain the discrepancy between the two studies. In addition, the fact that acrylamide was measured in heated flour rather than crisps, with the flour method producing higher levels of acrylamide, could potentially accentuate differences. We therefore reaffirm our advice that the conditions of potato storage must be carefully controlled to avoid exacerbating the risk of acrylamide formation during cooking and processing, and that potatoes should not be used for frying, baking or roasting beyond their optimum storage window, which differs between varieties.

From the Doncaster site, Harmony had the greatest acrylamide, while from the Woburn site, Pentland Dell, Lady Blanca, Harmony and Desiree had greatest acrylamide. After storage, the levels in Harmony from Woburn fell, but levels remained high in Lady Blanca, Pentland Dell, and Desiree, which had greatest acrylamide after storage for that site (see relevant values in bold in Table 2). In terms of producing low acrylamide, Lady Claire and Verdi, which have performed consistently well in previous studies (Elmore et al., 2015, Halford et al., 2012b, Muttucumaru et al., 2013, Muttucumaru et al., 2014b), were consistently low again in this study, particularly after the longer storage period. Lady Rosetta was one of the best varieties after the shorter storage period but its levels rose after the longer period, confirming it as a good variety to use from early storage (Halford et al., 2012b), while Hermes was the worst performer of the crisping varieties. Markies again showed good storage stability (*i.e.* its acrylamide-forming potential did not increase during storage) (Halford et al., 2012b) and another impressive French fry variety was Fontane, which was consistently low over both sites and storage periods.

**Fig. 2:**
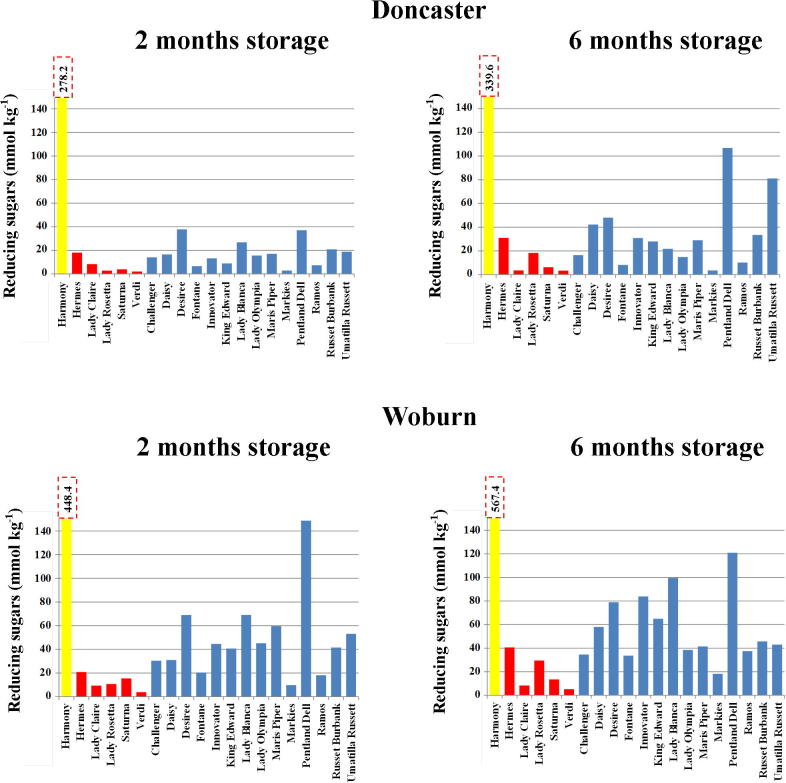
Mean concentrations of total reducing sugars (mmol kg^−1^ dry weight) (back-transformed means from analysis of variance) in twenty potato varieties grown at sites near Doncaster and Woburn, UK, and stored at 8 °C for two or six months as indicated. The boiling type (Harmony) is shown in yellow, crisping type in red and French fry type in blue. The columns for Harmony are truncated to allow them to be shown on the same scale as the others, and the actual figures are given above each column. For statistical analysis see Table 1, Table 2. (For interpretation of the references to colour in this figure legend, the reader is referred to the web version of this article.)

Harmony had by far the greatest concentration of reducing sugars (Fig. 2), though for this variety only fructose increased significantly (p < 0.05, LSD) with storage (Table 2). While the general trend was for increased sugars with longer storage (Fig. 2), the importance of the site effect was seen when this generalisation did not hold: for French fry varieties Maris Piper, Pentland Dell and Umatilla Russet from the Doncaster site, for example, glucose and fructose increased under longer storage, but for these varieties from the Woburn site the two reducing sugars fell after longer storage (Table 2). Of the crisping varieties, glucose and fructose in Lady Claire fell with longer storage for both sites, whereas for Saturna these sugars increased for Doncaster but decreased for Woburn under longer storage. The reason why potatoes of the same variety grown at different sites should behave differently in the same storage conditions is not known, but the fact that they do highlights the difficulty that the food industry has in predicting the acrylamide-forming potential of the potatoes it uses as its raw material (Powers et al., 2013).

**Fig. 3:**
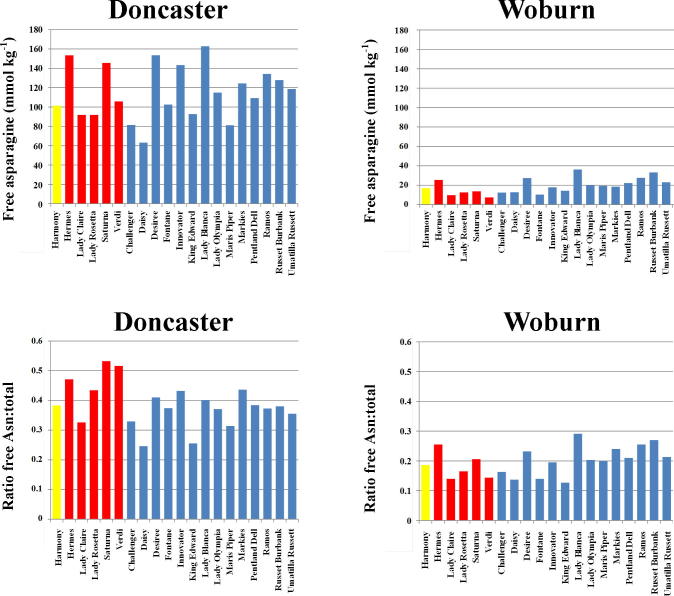
Mean free asparagine concentrations (top row) (mmol kg^−1^ dry weight) and the ratio of free asparagine to total free amino acids (bottom row) (back-transformed means from analysis of variance) in twenty potato varieties grown at sites near Doncaster and Woburn, UK, as indicated. The boiling type (Harmony) is shown in yellow, crisping type in red and French fry type in blue. For statistical analysis see Tables 1 and S2. (For interpretation of the references to colour in this figure legend, the reader is referred to the web version of this article.)

Free asparagine concentration provided the starkest contrast between the two sites, showing a significant (p < 0.05, F-tests) site by variety (nested in type) interaction (Tables 1 and S2) and, independently from this, a site by type by storage interaction (Tables 1 and S3). The ratio of free asparagine to total free amino acids also showed these interactions as well as a variety nested in type by storage interaction (Tables 1 and S4). The first of these results (Table S2) indicated that the varieties accumulated free asparagine differentially across the two sites, while the second (Table S3) indicated a differential effect of storage on free asparagine concentration for the different type (crisp, French fry and boiling) by site combinations such that storage had a general decreasing effect on asparagine, and significantly so (p < 0.05, LSD) for all types at Woburn but only for the French fry varieties at Doncaster. The third result (Table S4) shows that the varieties themselves responded differently to storage as regards the proportion of asparagine accumulated. The free asparagine and ratio of free asparagine to total free amino acids data from Table S2 are shown graphically in Fig. 3.

There was much greater free asparagine for all varieties grown at the Doncaster site compared with the Woburn site (Fig. 3). This was not just due to the Doncaster samples having a higher concentration of free amino acids, because the result was also reflected in the ratio of asparagine to total free amino acids (Fig. 3). This ratio had site by variety nested in type, site by type by storage, and variety nested within type by storage interactions (p < 0.05, F-tests) (Tables S2–S4). Hermes had the greatest ratio of free asparagine to total free amino acids before storage, but was superseded by Saturna, Lady Blanca and Russet Burbank after storage.

The fact that the potatoes from the two sites had different ratios of free asparagine to total free amino acids is important because free asparagine and total free amino acid concentrations have been shown to correlate significantly (*r* = 0.802, p < 0.001, F-test) in potatoes fertilised with nitrogen applied at rates of 0, 100 or 200 kg per hectare (Muttucumaru et al., 2014b), suggesting that the stepwise change in the ratio observed between the Doncaster and Woburn sites in the present study was not caused by differences in nitrogen availability arising from the different soil types.

Free asparagine is an important nitrogen transport molecule in many plant species but not in potato (Karley et al., 2002, Muttucumaru et al., 2014a). However, it accumulates in diverse plant species in response to a range of biotic and abiotic stresses (Lea, Sodek, Parry, Shewry, & Halford, 2007) and in a previous study has been shown to accumulate in the tubers of potatoes grown under severe drought stress under glass, although less severe drought stress led to an increase in free proline rather than free asparagine concentration (Muttucumaru et al., 2015). In that study, there was a significant negative correlation (*r* = −0.589, p < 0.001, F-test) between free proline concentration and acrylamide formation, consistent with proline having been shown to inhibit acrylamide formation in model systems (Koutsidis et al., 2009). However, the plots at both sites in this study were irrigated, and free proline was present in the potatoes at much lower concentrations than free asparagine, as is usually the case, and no significant (p < 0.05) negative correlation was observed (not shown). This was despite the fact that free proline concentration showed a significant site by variety nested within type by storage interaction (p < 0.001, F-test), and concentrations in the Doncaster potatoes were much higher than those in the Woburn potatoes (Table S1).

Other metabolites of interest included sucrose, which had variety nested within type by storage (Table S4), site by storage (Table S5) and site by variety nested in type interactions (Table S2), as did leucine, isoleucine, phenylalanine, tyrosine and tryptophan (p < 0.05, F-tests). Sucrose was higher for all varieties grown at Woburn than at Doncaster, except for Saturna and Umatilla Russet (Table S2). On average, crisp type Hermes had the greatest sucrose after 2 months’ storage (Table S4), while Lady Rosetta had greatest sucrose after 6 months (rising 1.4-fold from 2 to 6 months), significantly (p < 0.05, LSD) higher than all the other varieties apart from Lady Olympia, a variety that clearly retains a high sucrose concentration despite storage. Boiling type Harmony had by far the least sucrose, which, together with its very high concentrations of glucose and fructose, is consistent with a previous analysis of this variety and is suggestive of high invertase activity (Muttucumaru et al., 2014b). Overall, there was a trend for sucrose concentration to decrease during storage but this was significant (p < 0.05, LSD) only for the Woburn potatoes (Table S5).

There was also a full site by variety nested within type by storage interaction for free valine (p = 0.014, F-test), methionine (p = 0.033, F-test) and glutamic acid (p = 0.039, F-test) (Table S1). α-Aminobutyric acid (AABA) had variety nested within type by storage, site by variety nested in type and site by type by storage interactions (p < 0.05, F-tests) (Tables S2–S4). Free alanine, threonine, serine, glutamine, ornithine, lysine and total free amino acids had site by storage and a site by variety nested in type interactions (p < 0.05, F-tests) (Tables S2 and S5). Free aspartic acid and histidine had site by storage and variety nested within type effects (p < 0.05, F-tests) (Tables S5 and S6) that indicate a separation of the storage and site effects from the varietal contribution to variation. Free glycine had a site by variety nested within type interaction and a main effect of storage (p < 0.05, F-tests) (Tables S2 and S7), again indicating independence of effects. γ-Aminobutyric acid (GABA) had an effect of variety nested within type and a site by type interaction (p < 0.05, F-tests) (Tables S6 and S8), so was the only measured variable not affected in some way by storage.

### 3.2 The relationship between precursor concentration and acrylamide formation

Previous studies have generally found reducing sugar concentration to be the major determinant of acrylamide-forming potential in potato (Amrein et al., 2003, Becalski et al., 2004, De Wilde et al., 2005, Elmore et al., 2007, Elmore et al., 2010, Halford et al., 2012b, Muttucumaru et al., 2013, Muttucumaru et al., 2014b, Shepherd et al., 2010), but free asparagine concentration has been shown to contribute to the variance in acrylamide-forming potential in some studies, notably for French fry varieties (Halford et al., 2012b, Muttucumaru et al., 2013, Muttucumaru et al., 2014b, Shepherd et al., 2010), which are typically higher in reducing sugar concentration than crisping varieties.

**Fig. 4:**
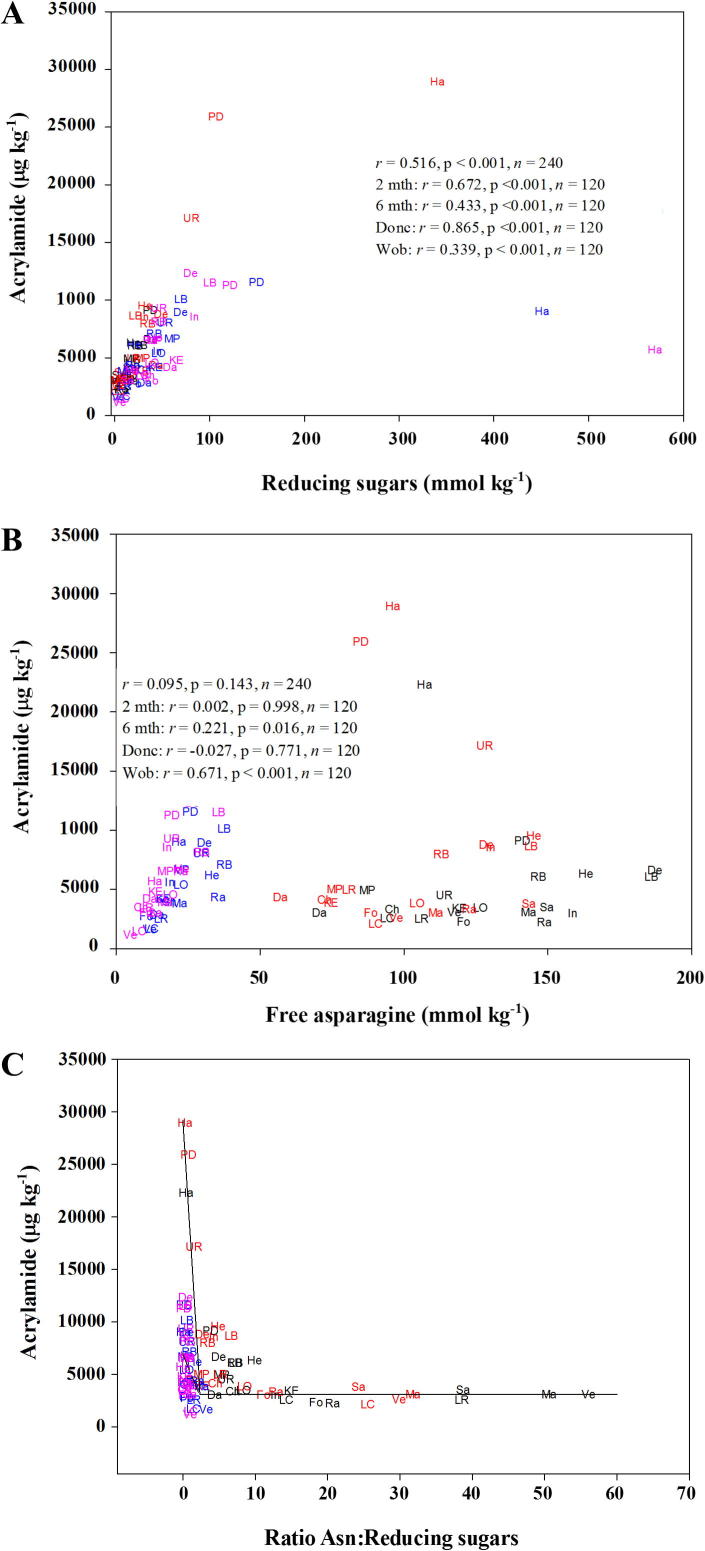
Graphs showing correlations between precursor concentration and acrylamide formation in heated (160 °C for 20 min) flour from twenty potato varieties grown at sites near Doncaster and Woburn, UK, and stored at 8 °C for two or six months. a. Reducing sugar concentration (mmol kg^−1^ dry weight) and acrylamide formation (μg kg^−1^ dry weight). b. Free asparagine concentration (mmol kg^−1^ dry weight) and acrylamide formation (μg kg^−1^ dry weight). c. Ratio of free asparagine to reducing sugar concentration and acrylamide formation (μg kg^−1^ dry weight). The varieties are plotted as two-letter codes: Challenger (Ch), Daisy (Da), Desiree (De), Fontane (Fo), Harmony (Ha), Hermes (He), Innovator (In), King Edward (KE), Lady Blanca (LB), Lady Claire (LC), Lady Olympia (LO), Lady Rosetta (LR), Maris Piper (MP), Markies (Ma), Pentland Dell (PD), Ramos (Ra), Russet Burbank (RB), Saturna (Sa) Umatilla Russet (UR) and Verdi (Ve); for Doncaster after two months storage (black) or six months storage (red), and Woburn after two months storage (blue) or six months storage (pink). The fitted model (Eq. (1)) is given as a solid line for the Doncaster potatoes and a dashed line for the Woburn potatoes. R^2^ = 60.4; standard error of observations = 3197 on 235 degrees of freedom.

The data from the present study revealed a significant (*r* = 0.516, p < 0.001, F-test) correlation between total reducing sugar concentration and acrylamide formation (Fig. 4a), with similar correlations between the individual reducing sugars, glucose or fructose, and acrylamide formation (not shown). However, the correlation coefficient was higher after 2 months’ storage than 6 months (*r* = 0.672 compared with 0.433) and there was a stark contrast in correlation coefficient between the sites, with that for the Doncaster site being considerably higher than that for the Woburn site (*r* = 0.865 compared with 0.339). Free asparagine concentration, on the other hand, correlated significantly, albeit weakly, with acrylamide formation overall only after 6 months’ storage (*r* = 0.221, p = 0.016, F-test) (Fig. 4b), but correlated significantly with acrylamide formation in the Woburn samples (*r* = 0.671, p < 0.001, F-test) regardless of storage, whereas it did not correlate significantly in the Doncaster samples (*r* = −0.027, p = 0.771, F-test).

These observations, together with those from previous studies, suggested that, while the relationship between acrylamide formation and reducing sugar concentration was stronger than that observed between acrylamide and free asparagine concentration, free asparagine concentration could contribute to the variance in acrylamide-forming potential when its concentration was relatively low compared with the concentration of reducing sugars. The ratio of asparagine to reducing sugars was therefore plotted against acrylamide formation. A roughly nonlinear, hyperbolic relationship was observed, but which could also be considered as two linear portions, *i.e.* in the form of a “broken stick” (Fig. 4c). For investigation, such a model was therefore fitted to the data, and took the form:

y=[(a+b∗Asn/RedSug]∗(Asn/RedSug<c)+[(a+(b∗c)+d∗Asn/RedSug]∗(Asn/RedSug>c)

where *y* is acrylamide, *a* is the intercept for the first linear portion, *b* is the slope of the first linear portion, *c* is the break-point in the model and *d* is the slope of the second linear portion. The model was fitted using ordinary least squares regression to estimate the parameters (*a*, *b*, *c* and *d*). The particular parameter of interest, *c*, indicates the value of the asparagine to reducing sugars ratio below which asparagine affects acrylamide formation. Further modelling tested (F-tests) the statistical significance of the effects of storage and site as regards the requirement of separate estimates of these parameters in the model.

Initial modelling revealed that the slope of the second portion (parameter *d*) was not significantly different from zero (p = 0.863, F-test) and so was fixed as such. Also, there was no effect of storage on any of the other parameters in the model with separately estimated values for each of them being non-significant (p = 0.075 for parameter *a*, p = 0.493 for *b*, and p = 0.978 for *c*, F-tests). There was, however, an effect of site for both *a* (p < 0.001, F-test) and *b* (p < 0.001, F-test) parameters, but crucially not for the *c* parameter (p = 0.998), there being no evidence of a second linear portion for the data from the Woburn site.

The fitted model was therefore:

(1) $$\begin{array}{cc} \text{Acrylamide}= & [29414-11138*(\text{Asn}/\text{Red Sugars})]*(\text{Asn}/\text{Red Sugars}<2.257)+\\ & \left[(29414-11138*2.257)]*(\text{Asn}/\text{Red Sugars}>2.257)\;(\text{Doncaster}\right)\\ & [6675-1061*(\text{Asn}/\text{Red Sugars})]*(\text{Asn}/\text{Red Sugars}<2.257)+\\ & \left[(29414-11138*2.257)]*(\text{Asn}/\text{Red Sugars}>2.257)\;(\text{Woburn}\right) \end{array}$$

The estimated parameters with standard errors (SEs) in brackets were: *a* (Doncaster) = 29414 (1420), *a* (Woburn) = 6675 (402), *b* (Doncaster) = −11138 (1158), *b* (Woburn) = −1061 (244) and *c* = 2.257 (0.149), and the level of acrylamide at *c* was estimated, using the model, to be 4279 (297). The model explained 59.7% (adjusted R^2^) of the variance in the data. These results suggest that there is a critical value of the asparagine to reducing sugars ratio, *i.e.* 2.257, below which free asparagine concentration contributes to the variance in acrylamide formation, and above which it is reducing sugar concentration alone that determines the formation of acrylamide. This would explain why the much higher levels of free asparagine in the Doncaster potatoes did not give rise to higher levels of acrylamide formation, because the ratio of free asparagine to reducing sugars in most of those potatoes was well above 2.257.

It is notable that cereal grains generally have a much lower concentration of free asparagine than potatoes, typically less than 10 mmol per kg (Curtis et al., 2009, Curtis et al., 2010, Granvogl et al., 2007, Muttucumaru et al., 2006, Postles et al., 2013), and a much lower ratio of free asparagine to reducing sugars, although wheat will accumulate free asparagine to concentrations comparable to those of potato if it is starved of sulphur (Curtis et al., 2009, Granvogl et al., 2007, Muttucumaru et al., 2006). Acrylamide formation in heated wheat and rye flour is determined by free asparagine concentration unless the flour is derived from sulphur-deprived wheat, consistent with the hypothesis that there is a tipping point in the ratio of free asparagine to reducing sugars below which free asparagine affects acrylamide formation and above which reducing sugar concentration becomes the determining factor.

The data and hypothesis presented here are also consistent with studies that have shown that acrylamide-forming potential can be reduced greatly by ribonucleic acid (RNA) interference-mediated suppression of asparagine synthetase activity in potato tubers (Chawla et al., 2012, Rommens et al., 2008). Those studies showed that free asparagine concentration would become limiting for acrylamide formation in potato if the concentration were low enough. Biotech varieties incorporating this trait were deregulated and cleared for commercial release in 2015. Importantly, the analyses reported here suggest that it is the ratio of free asparagine to reducing sugar concentration that should be targeted in order to reduce the acrylamide-forming potential of potatoes, rather than free asparagine concentration *per se*.

## 4 Conclusions

This study showed a clear effect of location of cultivation on the acrylamide-forming potential of potatoes, despite the fact that the two sites used in the study were only 120 miles apart. The potatoes from the two sites differed not only in composition but also in how that composition was affected by storage, highlighting the difficulty that the food industry has in achieving consistent regulatory compliance for the acrylamide content of its products while dealing with a raw material that is sometimes very unpredictable in its composition. The unusually wide range of ratios of free asparagine to reducing sugars in the dataset enabled modelling of the relationship between that parameter and acrylamide-forming potential and identified a value of 2.257 ± 0.149 as the tipping point in the ratio below which free asparagine concentration could affect acrylamide formation. This model could explain the apparent difference in the relationship between free asparagine concentration, reducing sugar concentration and acrylamide-forming potential in potato- and cereal-based food matrices.

## Figures and Tables

**Table 1:** Analysis of variance results: p-values for the main effects and interactions between the factors of site (S), type (T), variety (V) and storage (St), where the dot indicates the interaction. The common abbreviations for amino acids have been used. The most important (p < 0.05, F-test) terms for inspection given the ANOVA results are given in bold.

Variable	ANOVA term										
	S	T	S.T	T.V	S.T.V	St	S.St	T.St	S.T.St	T.V.St	S.T.V.St
Acrylamide	0.977	<0.001	<0.001	<0.001	<0.001	<0.001	<0.001	<0.001	0.156	<0.001	**0.003**

Sugars											
Glucose	<0.001	<0.001	0.007	<0.001	<0.001	<0.001	0.009	0.110	0.561	<0.001	**0.001**
Fructose	<0.001	<0.001	0.028	<0.001	<0.001	<0.001	0.007	0.215	0.249	<0.001	**<0.001**
Sucrose	<0.001	<0.001	0.052	<0.001	**<0.001**	<0.001	**0.007**	0.427	0.202	**<0.001**	0.170
Reducing	<0.001	<0.001	0.012	<0.001	<0.001	<0.001	0.004	0.571	0.565	<0.001	**<0.001**
Total Sugar	<0.001	<0.001	<0.001	<0.001	<0.001	<0.001	0.005	0.013	0.152	<0.001	**<0.001**

Amino Acids											
Alanine	0.003	<0.001	0.005	<0.001	**0.022**	<0.001	**<0.001**	0.189	0.055	0.154	0.800
Glycine	<0.001	<0.001	0.508	<0.001	**<0.001**	**<0.001**	0.398	0.219	0.263	0.091	0.110
AABA	<0.001	<0.001	0.017	<0.001	**<0.001**	<0.001	<0.001	<0.001	**0.005**	**0.005**	0.457
Valine	<0.001	<0.001	<0.001	<0.001	<0.001	<0.001	0.487	<0.001	0.876	<0.001	**0.014**
Leucine	<0.001	<0.001	0.008	<0.001	**<0.001**	<0.001	**0.006**	<0.001	0.674	**<0.001**	0.058
Isoleucine	<0.001	<0.001	0.840	<0.001	**<0.001**	<0.001	**<0.001**	<0.001	0.953	**<0.001**	0.140
Threonine	<0.001	<0.001	0.056	<0.001	**0.003**	0.051	**<0.001**	0.438	0.284	0.058	0.686
Serine	<0.001	<0.001	0.499	<0.001	**0.004**	0.176	**<0.001**	0.735	0.487	0.056	0.642
GABA	0.002	0.052	**0.014**	**<0.001**	0.067	0.500	0.888	0.851	0.393	0.582	0.817
Proline	<0.001	<0.001	<0.001	<0.001	<0.001	<0.001	<0.001	<0.001	0.139	0.076	**<0.001**
Asparagine	<0.001	<0.001	<0.001	<0.001	**<0.001**	<0.001	0.015	0.506	**0.002**	0.136	0.058
Aspartic acid	0.002	<0.001	0.430	**<0.001**	0.217	<0.001	**<0.001**	0.161	0.430	0.262	0.373
Methionine	<0.001	<0.001	0.983	<0.001	0.007	<0.001	0.546	0.055	0.675	<0.001	**0.033**
Glutamine	0.002	<0.001	0.535	<0.001	0.022	<0.001	<0.001	0.487	0.714	0.352	**0.039**
Phenylalanine	<0.001	<0.001	0.165	<0.001	**<0.001**	<0.001	**<0.001**	0.022	0.316	**<0.001**	0.055
Glutamine	<0.001	<0.001	0.182	<0.001	**0.005**	0.055	**0.005**	0.069	0.717	0.554	0.074
Ornithine	<0.001	<0.001	0.005	<0.001	**0.043**	0.628	**<0.001**	0.620	0.854	0.623	0.570
Lysine	0.707	<0.001	0.394	<0.001	**0.022**	0.002	**<0.001**	0.875	0.318	0.315	0.094
Histidine	0.026	<0.001	0.291	**0.005**	0.133	<0.001	**<0.001**	0.266	0.537	0.606	0.198
Tyrosine	0.234	0.001	0.043	<0.001	**0.001**	0.169	**<0.001**	0.226	0.416	**<0.001**	0.362
Tryptophan	0.002	0.082	0.005	<0.001	**<0.001**	0.003	**<0.001**	0.433	0.615	**<0.001**	0.140
Total	<0.001	<0.001	0.016	<0.001	**0.025**	<0.001	**<0.001**	0.141	0.203	0.057	0.086
Asn/Total	<0.001	0.031	<0.001	<0.001	**<0.001**	<0.001	0.040	0.010	**<0.001**	**0.008**	0.093

**Table 2:** Means tables showing site by variety nested in type by storage interaction for acrylamide formed in heated flour (μg kg^−1^), glucose, fructose and total reducing sugars (mmol kg^−1^) (*n* = 3). a. Log_e_ scale. b. Back-transformed means. Means of particular interest are given in bold or bold italics (see text).

a. Log_e_ scale																	
Type	Variety	Variable, Site and Storage															
		Acrylamide				Glucose				Fructose				Total Reducing Sugars			
		Doncaster		Woburn		Doncaster		Woburn		Doncaster		Woburn		Doncaster		Woburn	
		2	6	2	6	2	6	2	6	2	6	2	6	2	6	2	6
Boil	Harmony	**9.991**	**10.270**	**9.104**	8.637	**5.350**	**5.383**	**5.894**	**5.851**	**4.212**	**4.804**	**4.441**	**5.392**	**5.628**	**5.828**	**6.106**	**6.341**

Crisp	Hermes	8.735	9.144	8.719	8.757	2.345	2.800	2.409	3.120	2.033	2.674	2.275	2.888	2.894	3.433	3.037	3.704
	Lady Claire	7.828	7.631	7.404	7.246	1.479	0.648	1.605	1.485	1.372	0.479	1.461	1.319	2.123	1.261	2.230	2.102
	Lady Rosetta	7.823	8.472	7.823	8.127	0.436	2.081	1.686	2.693	0.197	2.310	1.661	2.692	1.019	2.907	2.367	3.386
	Saturna	8.134	8.207	8.335	7.997	0.649	1.273	2.194	2.007	0.663	0.965	1.858	1.808	1.355	1.825	2.734	2.606
	Verdi	7.979	7.853	7.387	7.028	0.239	0.684	0.670	1.143	-0.258	0.234	0.534	0.657	0.736	1.180	1.299	1.622

French fry	Challenger	8.102	8.307	8.261	8.116	2.166	2.214	2.945	2.973	1.677	1.991	2.425	2.709	2.646	2.803	3.412	3.544
	Daisy	7.991	8.365	7.943	8.330	2.359	3.143	2.841	3.414	1.786	2.946	2.612	3.318	2.807	3.742	3.429	4.060
	Desiree	8.785	9.067	**9.088**	**9.395**	3.040	3.248	3.762	3.825	2.825	3.106	3.257	3.499	3.632	3.873	4.234	4.368
	Fontane	7.716	7.998	7.890	7.962	1.420	1.512	2.534	3.013	0.908	1.261	2.052	2.588	1.892	2.088	3.015	3.516
	Innovator	7.955	9.040	***8.619***	***9.044***	2.101	2.867	3.371	3.957	1.633	2.585	2.733	3.447	2.590	3.429	3.796	4.428
	King Edward	7.955	8.254	8.326	8.421	1.679	2.650	3.271	3.594	1.232	2.630	2.642	3.350	2.191	3.334	3.705	4.172
	Lady Blanca	8.699	9.057	**9.204**	**9.324**	2.773	2.511	3.796	4.137	2.375	2.243	3.204	3.604	3.287	3.079	4.236	4.600
	Lady Olympia	8.131	8.181	8.570	8.402	2.277	2.199	3.339	3.155	1.773	1.779	2.826	2.709	2.749	2.705	3.808	3.650
	Maris Piper	8.478	8.500	8.786	8.768	2.277	2.762	3.576	3.137	1.982	2.575	3.173	2.916	2.834	3.366	4.088	3.726
	Markies	7.996	8.003	8.243	8.260	0.505	0.714	1.802	2.208	0.214	0.403	1.229	2.208	1.070	1.276	2.270	2.901
	Pentland Dell	**9.106**	**10.140**	**9.345**	**9.323**	3.071	4.069	4.515	4.262	2.743	3.878	4.049	3.911	3.614	4.671	5.002	4.795
	Ramos	7.672	8.052	8.364	8.776	1.304	1.718	2.393	2.996	1.300	1.535	1.967	2.860	1.999	2.327	2.898	3.624
	Russet Burbank	8.693	8.929	8.853	8.998	2.611	2.925	3.262	3.281	1.971	2.691	2.735	2.951	3.035	3.509	3.727	3.823
	Umatilla Russet	***8.355***	***9.710***	8.989	**9.095**	2.429	3.728	3.502	3.181	2.003	3.676	2.986	2.931	2.933	4.396	3.970	3.758

Within Site, Type and Storage SED (df)		0.1738 (151)				0.2724 (155)				0.2566 (155)				0.2556 (155)			
LSD (5%)		0.3433				0.5381				0.5068				0.5049			

Within Site, Type and Variety SED (df)		0.1619 (79)				0.2674 (80)				0.2558 (80)				0.2504 (80)			
LSD (5%)		0.3223				0.5321				0.5090				0.4983			

Other comparisons SED (df)		0.1760 (151)				0.2710 (159)				0.2561 (159)				0.2544 (159)			
LSD (5%)		0.3478				0.5352				0.5057				0.5024			

b. Back-transformed means																	
Type	Variety	Variable, Site and Storage															
		Acrylamide				Glucose				Fructose				Total Reducing sugars			
		Doncaster		Woburn		Doncaster		Woburn		Doncaster		Woburn		Doncaster		Woburn	
		2	6	2	6	2	6	2	6	2	6	2	6	2	6	2	6
Boil	Harmony	**21829**	**28854**	**8991**	5636	210.6	217.6	362.9	347.5	67.5	122.0	84.8	219.6	278.2	339.6	448.4	567.4

Crisp	Hermes	6217	9358	6118	6355	10.4	16.4	11.1	22.7	7.6	14.5	9.7	18.0	18.1	31.0	20.8	40.6
	Lady Claire	2510	2061	1643	1402	4.4	1.9	5.0	4.4	3.9	1.6	4.3	3.7	8.4	3.5	9.3	8.2
	Lady Rosetta	2497	4779	2497	3385	1.5	8.0	5.4	14.8	1.2	10.1	5.3	14.8	2.8	18.3	10.7	29.5
	Saturna	3408	3667	4167	2972	1.9	3.6	9.0	7.4	1.9	2.6	6.4	6.1	3.9	6.2	15.4	13.5
	Verdi	2919	2573	1615	1128	1.3	2.0	2.0	3.1	0.8	1.3	1.7	1.9	2.1	3.3	3.7	5.1

French fry	Challenger	3301	4052	3870	3348	8.7	9.2	19.0	19.6	5.4	7.3	11.3	15.0	14.1	16.5	30.3	34.6
	Daisy	2954	4294	2816	4146	10.6	23.2	17.1	30.4	6.0	19.0	13.6	27.6	16.6	42.2	30.9	58.0
	Desiree	6535	8665	**8848**	**12028**	20.9	25.7	43.0	45.8	16.9	22.3	26.0	33.1	37.8	48.1	69.0	78.9
	Fontane	2244	2975	2670	2870	4.1	4.5	12.6	20.3	2.5	3.5	7.8	13.3	6.6	8.1	20.4	33.7
	Innovator	2850	8434	**5536**	**8468**	8.2	17.6	29.1	52.3	5.1	13.3	15.4	31.4	13.3	30.9	44.5	83.8
	King Edward	2850	3843	4130	4541	5.4	14.2	26.3	36.4	3.4	13.9	14.0	28.5	8.9	28.0	40.7	64.9
	Lady Blanca	5997	8578	**9937**	**11204**	16.0	12.3	44.5	62.6	10.8	9.4	24.6	36.8	26.8	21.7	69.1	99.5
	Lady Olympia	3398	3572	5271	4456	9.7	9.0	28.2	23.4	5.9	5.9	16.9	15.0	15.6	14.9	45.1	38.5
	Maris Piper	4808	4915	6542	6425	9.7	15.8	35.7	23.0	7.3	13.1	23.9	18.5	17.0	29.0	59.6	41.5
	Markies	2969	2990	3801	3866	1.7	2.0	6.1	9.1	1.2	1.5	3.4	9.1	2.9	3.6	9.7	18.2
	Pentland Dell	**9009**	**25336**	**11441**	**11193**	21.6	58.5	91.4	71.0	15.5	48.3	57.3	49.9	37.1	106.8	148.7	120.9
	Ramos	2147	3140	4290	6477	3.7	5.6	11.0	20.0	3.7	4.6	7.2	17.5	7.4	10.2	18.1	37.5
	Russet Burbank	5961	7548	6995	8087	13.6	18.6	26.1	26.6	7.2	14.8	15.4	19.1	20.8	33.4	41.5	45.7
	Umatilla Russet	**4251**	**16482**	8014	8911	11.4	41.6	33.2	24.1	7.4	39.5	19.8	18.8	18.8	81.1	53.0	42.9
