# Supplementary material for: Acrylamide-forming potential of potatoes grown at different locations, and the ratio of free asparagine to reducing sugars at which free asparagine becomes a limiting factor for acrylamide formation
Source: Food Chem. 2017 Apr 1;220:76–86. doi: 10.1016/j.foodchem.2016.09.199 (PMC5119237; doi:10.1016/j.foodchem.2016.09.199)
Supplement: Supplementary data 2 [file mmc2.docx]

Acrylamide-forming potential of potatoes grown at different locations, and the ratio of free asparagine to reducing sugars at which free asparagine becomes a limiting factor for acrylamide formation

Nira Muttucumaru^1^, Stephen J. Powers^2^, J. Stephen Elmore^3^, Andrew Dodson^3^, Adrian Briddon^4^, Donald S. Mottram^3^ and Nigel G. Halford^1^

^1^Plant Biology and Crop Science Department, Rothamsted Research, Harpenden, Hertfordshire AL5 2JQ, United Kingdom

^2^Computational and Systems Biology Department, Rothamsted Research, Harpenden, Hertfordshire AL5 2JQ, United Kingdom

^3^Department of Food and Nutritional Sciences, University of Reading, Whiteknights, Reading, RG6 6AP, UK

^4^AHDB Potatoes, Sutton Bridge Crop Storage Research, East Bank, Sutton Bridge, Spalding, Lincolnshire PE12 9YD, United Kingdom

Correspondence: Nigel G. Halford, Plant Biology and Crop Science Department, Rothamsted Research, Harpenden, Hertfordshire, United Kingdom

Telephone: (44) (0) 1582 763133; Fax (44) (0) 1582 763010; Mobile: 44 785 762 6662

E-mail: [nigel.halford@rothamsted.ac.uk](mailto:nigel.halford@rothamsted.ac.uk)

Supplementary File S2

**Supplementary File S2**

Results of analysis of variance (ANOVA) of amino acid concentration (mmol kg^-1^), acrylamide formed in heated flour (µg kg^-1^) and sugar concentration (glucose, fructose and sucrose) (mmol kg^-1^) in samples of 20 potato varieties grown at two sites (Doncaster and Woburn) in randomised block designs with three blocks in 2011. The potatoes were harvested and transferred to storage, then sampled after 2 and 6 months. There were 240 observations for each response, comprising a two sites by 20 varieties by two storage conditions factorial treatment structure with three replicates. Also, the varieties were grouped into crisping, French fry and boiling type, to consider this aspect in analysis.

p-Values for the main effects and interactions between the terms of site (S), type (T), variety (V) and storage (St) are given in Table 1 of the main manuscript, and means tables for interpretation given the results in Table 1 are provided below in Tables S1-S8.

Table S1. Means tables for variables with site by variety nested in type by storage (2 or 6 months) interaction (*n* = 3)

a. Log_e_ scale

| Type | Variety | Variable, Site and Storage | | | | | | | | | | | |
| --- | --- | --- | --- | --- | --- | --- | --- | --- | --- | --- | --- | --- | --- |
|  |  | Acrylamide | | | | Glucose | | | | Fructose | | | |
|  |  | Doncaster | | Woburn | | Doncaster | | Woburn | | Doncaster | | Woburn | |
|  |  | 2 | 6 | 2 | 6 | 2 | 6 | 2 | 6 | 2 | 6 | 2 | 6 |
| Boil | Harmony | **9.991** | **10.270** | **9.104** | 8.637 | **5.350** | **5.383** | **5.894** | **5.851** | **4.212** | **4.804** | **4.441** | **5.392** |
| Crisp | Hermes | 8.735 | 9.144 | 8.719 | 8.757 | 2.345 | 2.800 | 2.409 | 3.120 | 2.033 | 2.674 | 2.275 | 2.888 |
|  | Lady Claire | 7.828 | 7.631 | 7.404 | 7.246 | 1.479 | 0.648 | 1.605 | 1.485 | 1.372 | 0.479 | 1.461 | 1.319 |
|  | Lady Rosetta | 7.823 | 8.472 | 7.823 | 8.127 | 0.436 | 2.081 | 1.686 | 2.693 | 0.197 | 2.310 | 1.661 | 2.692 |
|  | Saturna | 8.134 | 8.207 | 8.335 | 7.997 | 0.649 | 1.273 | 2.194 | 2.007 | 0.663 | 0.965 | 1.858 | 1.808 |
|  | Verdi | 7.979 | 7.853 | 7.387 | 7.028 | 0.239 | 0.684 | 0.670 | 1.143 | -0.258 | 0.234 | 0.534 | 0.657 |
| French fry | Challenger | 8.102 | 8.307 | 8.261 | 8.116 | 2.166 | 2.214 | 2.945 | 2.973 | 1.677 | 1.991 | 2.425 | 2.709 |
|  | Daisy | 7.991 | 8.365 | 7.943 | 8.330 | 2.359 | 3.143 | 2.841 | 3.414 | 1.786 | 2.946 | 2.612 | 3.318 |
|  | Desiree | 8.785 | 9.067 | **9.088** | **9.395** | 3.040 | 3.248 | 3.762 | 3.825 | 2.825 | 3.106 | 3.257 | 3.499 |
|  | Fontane | 7.716 | 7.998 | 7.890 | 7.962 | 1.420 | 1.512 | 2.534 | 3.013 | 0.908 | 1.261 | 2.052 | 2.588 |
|  | Innovator | 7.955 | 9.040 | **8.619** | **9.044** | 2.101 | 2.867 | 3.371 | 3.957 | 1.633 | 2.585 | 2.733 | 3.447 |
|  | King Edward | 7.955 | 8.254 | 8.326 | 8.421 | 1.679 | 2.650 | 3.271 | 3.594 | 1.232 | 2.630 | 2.642 | 3.350 |
|  | Lady Blanca | 8.699 | 9.057 | **9.204** | **9.324** | 2.773 | 2.511 | 3.796 | 4.137 | 2.375 | 2.243 | 3.204 | 3.604 |
|  | Lady Olympia | 8.131 | 8.181 | 8.570 | 8.402 | 2.277 | 2.199 | 3.339 | 3.155 | 1.773 | 1.779 | 2.826 | 2.709 |
|  | Maris Piper | 8.478 | 8.500 | 8.786 | 8.768 | 2.277 | 2.762 | 3.576 | 3.137 | 1.982 | 2.575 | 3.173 | 2.916 |
|  | Markies | 7.996 | 8.003 | 8.243 | 8.260 | 0.505 | 0.714 | 1.802 | 2.208 | 0.214 | 0.403 | 1.229 | 2.208 |
|  | Pentland Dell | **9.106** | **10.140** | **9.345** | **9.323** | 3.071 | 4.069 | 4.515 | 4.262 | 2.743 | 3.878 | 4.049 | 3.911 |
|  | Ramos | 7.672 | 8.052 | 8.364 | 8.776 | 1.304 | 1.718 | 2.393 | 2.996 | 1.300 | 1.535 | 1.967 | 2.860 |
|  | Russet Burbank | 8.693 | 8.929 | 8.853 | 8.998 | 2.611 | 2.925 | 3.262 | 3.281 | 1.971 | 2.691 | 2.735 | 2.951 |
|  | Umatilla Russet | **8.355** | **9.710** | 8.989 | **9.095** | 2.429 | 3.728 | 3.502 | 3.181 | 2.003 | 3.676 | 2.986 | 2.931 |
| Within Site, Type and Storage SED (df) | | 0.1738 (151) | | | | 0.2724 (155) | | | | 0.2566 (155) | | | |
| LSD (5%) | | 0.3433 | | | | 0.5381 | | | | 0.5068 | | | |
| Within Site, Type and Variety SED (df) | | 0.1619 (79) | | | | 0.2674 (80) | | | | 0.2558 (80) | | | |
| LSD (5%) | | 0.3223 | | | | 0.5321 | | | | 0.5090 | | | |
| Other comparisons SED (df) | | 0.1760 (151) | | | | 0.2710 (159) | | | | 0.2561 (159) | | | |
| LSD (5%) | | 0.3478 | | | | 0.5352 | | | | 0.5057 | | | |

Continued:

| Type | Variety | Variable, Site and Storage | | | | | | | | | | | |
| --- | --- | --- | --- | --- | --- | --- | --- | --- | --- | --- | --- | --- | --- |
|  |  | Total Reducing Sugars | | | | Total Sugars | | | | Glutamic acid | | | |
|  |  | Doncaster | | Woburn | | Doncaster | | Woburn | | Doncaster | | Woburn | |
|  |  | 2 | 6 | 2 | 6 | 2 | 6 | 2 | 6 | 2 | 6 | 2 | 6 |
| Boil | Harmony | **5.628** | **5.828** | **6.106** | **6.341** | **5.639** | **5.840** | **6.119** | **6.345** | 2.828 | 2.498 | 2.667 | 1.677 |
| Crisp | Hermes | 2.894 | 3.433 | 3.037 | 3.704 | 3.736 | 3.821 | 3.874 | 4.207 | 2.665 | 2.777 | 2.907 | 1.955 |
|  | Lady Claire | 2.123 | 1.261 | 2.230 | 2.102 | 3.102 | 2.505 | 3.303 | 2.848 | 3.239 | 2.998 | 2.933 | 2.748 |
|  | Lady Rosetta | 1.019 | 2.907 | 2.367 | 3.386 | 2.954 | 3.778 | 3.328 | 3.981 | 2.966 | 2.637 | 2.989 | 1.769 |
|  | Saturna | 1.355 | 1.825 | 2.734 | 2.606 | 3.176 | 3.077 | 3.532 | 3.308 | 3.225 | 2.918 | 2.365 | 2.157 |
|  | Verdi | 0.736 | 1.180 | 1.299 | 1.622 | 2.856 | 2.815 | 3.191 | 3.070 | 3.456 | 3.050 | 2.843 | 2.187 |
| French fry | Challenger | 2.646 | 2.803 | 3.412 | 3.544 | 3.310 | 3.421 | 3.926 | 3.971 | 3.026 | 2.638 | 2.532 | 1.965 |
|  | Daisy | 2.807 | 3.742 | 3.429 | 4.060 | 3.517 | 4.024 | 3.942 | 4.290 | 2.125 | 2.386 | 2.505 | 1.398 |
|  | Desiree | 3.632 | 3.873 | 4.234 | 4.368 | 3.986 | 4.204 | 4.518 | 4.607 | 3.211 | 2.780 | 3.098 | 1.840 |
|  | Fontane | 1.892 | 2.088 | 3.015 | 3.516 | 2.951 | 3.109 | 3.690 | 3.954 | 3.099 | 2.877 | 2.637 | 2.537 |
|  | Innovator | 2.590 | 3.429 | 3.796 | 4.428 | 3.309 | 3.918 | 4.101 | 4.654 | 3.414 | 3.132 | 2.501 | 2.398 |
|  | King Edward | 2.191 | 3.334 | 3.705 | 4.172 | 3.044 | 3.561 | 4.010 | 4.304 | 3.258 | 2.996 | 2.729 | 2.134 |
|  | Lady Blanca | 3.287 | 3.079 | 4.236 | 4.600 | 3.621 | 3.443 | 4.430 | 4.775 | 3.654 | 2.873 | 2.623 | 2.232 |
|  | Lady Olympia | 2.749 | 2.705 | 3.808 | 3.650 | 3.375 | 3.484 | 4.337 | 4.213 | 3.248 | 2.964 | 2.704 | 2.307 |
|  | Maris Piper | 2.834 | 3.366 | 4.088 | 3.726 | 3.491 | 3.619 | 4.410 | 3.900 | 3.119 | 2.877 | 2.756 | 2.235 |
|  | Markies | 1.070 | 1.276 | 2.270 | 2.901 | 2.941 | 2.705 | 3.277 | 3.494 | 3.101 | 3.152 | 2.975 | 2.225 |
|  | Pentland Dell | 3.614 | 4.671 | 5.002 | 4.795 | 3.914 | 4.861 | 5.120 | 4.937 | 3.275 | 2.950 | 2.788 | 2.265 |
|  | Ramos | 1.999 | 2.327 | 2.898 | 3.624 | 2.992 | 3.115 | 3.627 | 3.990 | 3.496 | 3.089 | 2.887 | 2.074 |
|  | Russet Burbank | 3.035 | 3.509 | 3.727 | 3.823 | 3.615 | 3.838 | 4.133 | 4.086 | 3.071 | 2.868 | 3.041 | 2.093 |
|  | Umatilla Russet | 2.933 | 4.396 | 3.970 | 3.758 | 3.498 | 4.611 | 4.241 | 4.060 | 3.016 | 2.911 | 2.772 | 2.177 |
| Within Site, Type and Storage SED (df) | | 0.2556 (155) | | | | 0.1335 (155) | | | | 0.2231 (146) | | | |
| LSD (5%) | | 0.5049 | | | | 0.2637 | | | | 0.4408 | | | |
| Within Site, Type and Variety SED (df) | | 0.2504 (80) | | | | 0.1320 (80) | | | | 0.2530 (80) | | | |
| LSD (5%) | | 0.4983 | | | | 0.2626 | | | | 0.5035 | | | |
| Other comparisons SED (df) | | 0.2544 (159) | | | | 0.1338 (159) | | | | 0.2352 (113) | | | |
| LSD (5%) | | 0.5024 | | | | 0.2642 | | | |  | | | |

Continued:

| Type | Variety | Variable and Site | | | | | | | | | | | |
| --- | --- | --- | --- | --- | --- | --- | --- | --- | --- | --- | --- | --- | --- |
|  |  | Valine | | | | Proline | | | | Methionine | | | |
|  |  | Doncaster | | Woburn | | Doncaster | | Woburn | | Doncaster | | Woburn | |
|  |  | 2 | 6 | 2 | 6 | 2 | 6 | 2 | 6 | 2 | 6 | 2 | 6 |
| Boil | Harmony | 2.479 | 2.575 | 1.723 | 1.797 | 1.465 | 1.606 | 0.194 | 0.833 | 0.822 | 0.470 | 0.081 | -0.513 |
| Crisp | Hermes | 2.553 | 2.356 | 1.396 | 1.331 | 1.724 | 1.669 | -0.012 | 0.902 | 0.680 | 0.078 | -0.239 | -1.360 |
|  | Lady Claire | 2.480 | 2.419 | 0.891 | 0.944 | 1.260 | 1.133 | -0.618 | -0.197 | 0.790 | 0.314 | -0.705 | -0.966 |
|  | Lady Rosetta | 1.889 | 1.882 | 1.217 | 1.240 | 2.478 | 2.162 | 0.154 | 0.884 | 0.011 | -0.354 | -0.611 | -0.664 |
|  | Saturna | 2.100 | 2.094 | 0.973 | 0.897 | 1.570 | 1.661 | -0.488 | 0.430 | 0.074 | -0.463 | -0.958 | -0.866 |
|  | Verdi | 1.428 | 1.571 | 0.448 | 0.124 | 1.157 | 1.366 | -0.171 | 0.510 | -1.026 | -0.406 | -0.997 | -1.538 |
| French fry | Challenger | 2.217 | 2.007 | 1.211 | 1.022 | 1.413 | 1.279 | -0.330 | 0.164 | 0.769 | 0.213 | -0.253 | -1.097 |
|  | Daisy | 2.874 | 2.823 | 1.935 | 1.921 | 1.785 | 1.630 | -0.083 | 0.516 | 1.535 | 1.374 | 0.633 | 0.252 |
|  | Desiree | 3.136 | 2.956 | 2.021 | 1.953 | 1.555 | 1.469 | 0.159 | 0.833 | 1.358 | 0.821 | 0.287 | -0.195 |
|  | Fontane | 2.557 | 2.383 | 1.210 | 1.233 | 2.413 | 1.987 | -0.471 | 0.158 | 0.390 | 0.010 | -0.614 | -0.384 |
|  | Innovator | 2.105 | 2.066 | 1.642 | 1.620 | 1.965 | 1.745 | 0.044 | 0.734 | 0.811 | 0.189 | 0.204 | -0.252 |
|  | King Edward | 2.953 | 2.826 | 2.021 | 2.125 | 2.887 | 1.915 | 0.240 | 1.166 | 1.331 | 0.751 | 0.232 | -0.122 |
|  | Lady Blanca | 2.958 | 2.722 | 2.281 | 2.090 | 2.139 | 1.787 | 0.306 | 0.758 | 0.618 | 0.122 | 0.348 | -0.520 |
|  | Lady Olympia | 2.926 | 2.946 | 2.037 | 2.236 | 2.114 | 2.077 | 0.594 | 1.243 | 0.936 | 0.511 | 0.368 | -0.021 |
|  | Maris Piper | 2.617 | 2.533 | 1.818 | 1.565 | 1.482 | 1.490 | 0.124 | 0.265 | 1.284 | 0.913 | 0.539 | 0.166 |
|  | Markies | 2.453 | 2.186 | 0.938 | 0.821 | 2.037 | 1.774 | -0.472 | 0.350 | 1.086 | 0.899 | -0.257 | -0.448 |
|  | Pentland Dell | 2.628 | 2.530 | 1.813 | 1.461 | 2.443 | 1.921 | 0.375 | 0.934 | 1.122 | 0.210 | 0.017 | -0.688 |
|  | Ramos | 2.944 | 2.645 | 1.988 | 1.340 | 3.045 | 2.579 | 0.040 | 0.572 | 1.433 | 1.086 | 0.714 | 0.157 |
|  | Russet Burbank | 2.978 | 2.728 | 2.078 | 1.826 | 2.155 | 1.833 | -0.126 | 0.468 | 1.317 | 0.617 | 0.473 | -0.658 |
|  | Umatilla Russet | 2.881 | 2.680 | 2.116 | 1.522 | 2.647 | 2.620 | 0.171 | 0.666 | 1.220 | 0.186 | 0.263 | -0.627 |
| Within Site, Type and Storage SED (df) | | 0.1225 (142) | | | | 0.1856 (127) | | | | 0.2517 (146) | | | |
| LSD (5%) | | 0.2421 | | | | 0.3673 | | | | 0.4975 | | | |
| Within Site, Type and Variety SED (df) | | 0.1040 (80) | | | | 0.1375 (80) | | | | 0.2217 (80) | | | |
| LSD (5%) | | 0.2069 | | | | 0.2737 | | | | 0.4412 | | | |
| Other comparisons SED (df) | | 0.1295 (102) | | | | 0.1929 (110) | | | | 0.2590 (134) | | | |
| LSD (5%) | | 0.2569 | | | | 0.3823 | | | | 0.5122 | | | |

b. Back-transformed means:

| Type | Variety | Variable, Site and Storage | | | | | | | | | | | |
| --- | --- | --- | --- | --- | --- | --- | --- | --- | --- | --- | --- | --- | --- |
|  |  | Acrylamide | | | | Glucose | | | | Fructose | | | |
|  |  | Doncaster | | Woburn | | Doncaster | | Woburn | | Doncaster | | Woburn | |
|  |  | 2 | 6 | 2 | 6 | 2 | 6 | 2 | 6 | 2 | 6 | 2 | 6 |
| Boil | Harmony | **21829** | **28854** | **8991** | 5636 | 210.6 | 217.6 | 362.9 | 347.5 | 67.5 | 122.0 | 84.8 | 219.6 |
| Crisp | Hermes | 6217 | 9358 | 6118 | 6355 | 10.4 | 16.4 | 11.1 | 22.7 | 7.6 | 14.5 | 9.7 | 18.0 |
|  | Lady Claire | 2510 | 2061 | 1643 | 1402 | 4.4 | 1.9 | 5.0 | 4.4 | 3.9 | 1.6 | 4.3 | 3.7 |
|  | Lady Rosetta | 2497 | 4779 | 2497 | 3385 | 1.5 | 8.0 | 5.4 | 14.8 | 1.2 | 10.1 | 5.3 | 14.8 |
|  | Saturna | 3408 | 3667 | 4167 | 2972 | 1.9 | 3.6 | 9.0 | 7.4 | 1.9 | 2.6 | 6.4 | 6.1 |
|  | Verdi | 2919 | 2573 | 1615 | 1128 | 1.3 | 2.0 | 2.0 | 3.1 | 0.8 | 1.3 | 1.7 | 1.9 |
| French fry | Challenger | 3301 | 4052 | 3870 | 3348 | 8.7 | 9.2 | 19.0 | 19.6 | 5.4 | 7.3 | 11.3 | 15.0 |
|  | Daisy | 2954 | 4294 | 2816 | 4146 | 10.6 | 23.2 | 17.1 | 30.4 | 6.0 | 19.0 | 13.6 | 27.6 |
|  | Desiree | 6535 | 8665 | **8848** | **12028** | 20.9 | 25.7 | 43.0 | 45.8 | 16.9 | 22.3 | 26.0 | 33.1 |
|  | Fontane | 2244 | 2975 | 2670 | 2870 | 4.1 | 4.5 | 12.6 | 20.3 | 2.5 | 3.5 | 7.8 | 13.3 |
|  | Innovator | 2850 | 8434 | **5536** | **8468** | 8.2 | 17.6 | 29.1 | 52.3 | 5.1 | 13.3 | 15.4 | 31.4 |
|  | King Edward | 2850 | 3843 | 4130 | 4541 | 5.4 | 14.2 | 26.3 | 36.4 | 3.4 | 13.9 | 14.0 | 28.5 |
|  | Lady Blanca | 5997 | 8578 | **9937** | **11204** | 16.0 | 12.3 | 44.5 | 62.6 | 10.8 | 9.4 | 24.6 | 36.8 |
|  | Lady Olympia | 3398 | 3572 | 5271 | 4456 | 9.7 | 9.0 | 28.2 | 23.4 | 5.9 | 5.9 | 16.9 | 15.0 |
|  | Maris Piper | 4808 | 4915 | 6542 | 6425 | 9.7 | 15.8 | 35.7 | 23.0 | 7.3 | 13.1 | 23.9 | 18.5 |
|  | Markies | 2969 | 2990 | 3801 | 3866 | 1.7 | 2.0 | 6.1 | 9.1 | 1.2 | 1.5 | 3.4 | 9.1 |
|  | Pentland Dell | **9009** | **25336** | **11441** | **11193** | 21.6 | 58.5 | 91.4 | 71.0 | 15.5 | 48.3 | 57.3 | 49.9 |
|  | Ramos | 2147 | 3140 | 4290 | 6477 | 3.7 | 5.6 | 11.0 | 20.0 | 3.7 | 4.6 | 7.2 | 17.5 |
|  | Russet Burbank | 5961 | 7548 | 6995 | 8087 | 13.6 | 18.6 | 26.1 | 26.6 | 7.2 | 14.8 | 15.4 | 19.1 |
|  | Umatilla Russet | **4251** | **16482** | 8014 | 8911 | 11.4 | 41.6 | 33.2 | 24.1 | 7.4 | 39.5 | 19.8 | 18.8 |

Continued:

| Type | Variety | Variable, Site and Storage | | | | | | | | | | | |
| --- | --- | --- | --- | --- | --- | --- | --- | --- | --- | --- | --- | --- | --- |
|  |  | Total Reducing sugars | | | | Total Sugars | | | | Glutamic acid | | | |
|  |  | Doncaster | | Woburn | | Doncaster | | Woburn | | Doncaster | | Woburn | |
|  |  | 2 | 6 | 2 | 6 | 2 | 6 | 2 | 6 | 2 | 6 | 2 | 6 |
| Boil | Harmony | 278.2 | 339.6 | 448.4 | 567.4 | 281.3 | 343.6 | 454.5 | 569.5 | 16.907 | 12.153 | 14.392 | 5.344 |
| Crisp | Hermes | 18.1 | 31.0 | 20.8 | 40.6 | 41.9 | 45.6 | 48.1 | 67.1 | 14.363 | 16.066 | 18.297 | 7.059 |
|  | Lady Claire | 8.4 | 3.5 | 9.3 | 8.2 | 22.3 | 12.2 | 27.2 | 17.3 | 25.503 | 20.040 | 18.779 | 15.606 |
|  | Lady Rosetta | 2.8 | 18.3 | 10.7 | 29.5 | 19.2 | 43.7 | 27.9 | 53.6 | 19.409 | 13.966 | 19.861 | 5.860 |
|  | Saturna | 3.9 | 6.2 | 15.4 | 13.5 | 24.0 | 21.7 | 34.2 | 27.3 | 25.149 | 18.499 | 10.639 | 8.640 |
|  | Verdi | 2.1 | 3.3 | 3.7 | 5.1 | 17.4 | 16.7 | 24.3 | 21.5 | 31.685 | 21.110 | 17.162 | 8.903 |
| French fry | Challenger | 14.1 | 16.5 | 30.3 | 34.6 | 27.4 | 30.6 | 50.7 | 53.0 | 20.610 | 13.980 | 12.574 | 7.130 |
|  | Daisy | 16.6 | 42.2 | 30.9 | 58.0 | 33.7 | 55.9 | 51.5 | 73.0 | 8.368 | 10.865 | 12.239 | 4.042 |
|  | Desiree | 37.8 | 48.1 | 69.0 | 78.9 | 53.9 | 67.0 | 91.7 | 100.2 | 24.799 | 16.114 | 22.149 | 6.292 |
|  | Fontane | 6.6 | 8.1 | 20.4 | 33.7 | 19.1 | 22.4 | 40.0 | 52.2 | 22.171 | 17.756 | 13.966 | 12.637 |
|  | Innovator | 13.3 | 30.9 | 44.5 | 83.8 | 27.4 | 50.3 | 60.4 | 105.0 | 30.382 | 22.915 | 12.190 | 10.996 |
|  | King Edward | 8.9 | 28.0 | 40.7 | 64.9 | 21.0 | 35.2 | 55.1 | 74.0 | 25.992 | 20.000 | 15.313 | 8.444 |
|  | Lady Blanca | 26.8 | 21.7 | 69.1 | 99.5 | 37.4 | 31.3 | 83.9 | 118.6 | 38.624 | 17.685 | 13.772 | 9.313 |
|  | Lady Olympia | 15.6 | 14.9 | 45.1 | 38.5 | 29.2 | 32.6 | 76.5 | 67.5 | 25.734 | 19.370 | 14.934 | 10.039 |
|  | Maris Piper | 17.0 | 29.0 | 59.6 | 41.5 | 32.8 | 37.3 | 82.2 | 49.4 | 22.619 | 17.756 | 15.732 | 9.341 |
|  | Markies | 2.9 | 3.6 | 9.7 | 18.2 | 18.9 | 14.9 | 26.5 | 32.9 | 22.215 | 23.378 | 19.585 | 9.248 |
|  | Pentland Dell | 37.1 | 106.8 | 148.7 | 120.9 | 50.1 | 129.1 | 167.3 | 139.4 | 26.438 | 19.101 | 16.243 | 9.626 |
|  | Ramos | 7.4 | 10.2 | 18.1 | 37.5 | 19.9 | 22.5 | 37.6 | 54.1 | 32.978 | 21.950 | 17.934 | 7.952 |
|  | Russet Burbank | 20.8 | 33.4 | 41.5 | 45.7 | 37.1 | 46.4 | 62.3 | 59.5 | 21.558 | 17.597 | 20.921 | 8.104 |
|  | Umatilla Russet | 18.8 | 81.1 | 53.0 | 42.9 | 33.1 | 100.5 | 69.4 | 58.0 | 20.404 | 18.370 | 15.986 | 8.815 |

Continued:

| Type | Variety | Variable and Site | | | | | | | | | | | |
| --- | --- | --- | --- | --- | --- | --- | --- | --- | --- | --- | --- | --- | --- |
|  |  | Valine | | | | Proline | | | | Methionine | | | |
|  |  | Doncaster | | Woburn | | Doncaster | | Woburn | | Doncaster | | Woburn | |
|  |  | 2 | 6 | 2 | 6 | 2 | 6 | 2 | 6 | 2 | 6 | 2 | 6 |
| Boil | Harmony | 11.924 | 13.126 | 5.596 | 6.027 | 4.323 | 4.978 | 1.209 | 2.295 | 2.270 | 1.595 | 1.079 | 0.594 |
| Crisp | Hermes | 12.841 | 10.544 | 4.034 | 3.780 | 5.602 | 5.302 | 0.983 | 2.460 | 1.969 | 1.076 | 0.782 | 0.252 |
|  | Lady Claire | 11.936 | 11.230 | 2.433 | 2.565 | 3.520 | 3.100 | 0.534 | 0.816 | 2.198 | 1.364 | 0.489 | 0.376 |
|  | Lady Rosetta | 6.608 | 6.562 | 3.372 | 3.451 | 11.912 | 8.683 | 1.161 | 2.416 | 1.006 | 0.697 | 0.538 | 0.510 |
|  | Saturna | 8.161 | 8.112 | 2.641 | 2.447 | 4.802 | 5.260 | 0.609 | 1.532 | 1.072 | 0.624 | 0.379 | 0.416 |
|  | Verdi | 4.165 | 4.806 | 1.560 | 1.127 | 3.175 | 3.915 | 0.838 | 1.660 | 0.353 | 0.661 | 0.364 | 0.210 |
| French fry | Challenger | 9.175 | 7.436 | 3.352 | 2.774 | 4.103 | 3.588 | 0.714 | 1.173 | 2.153 | 1.232 | 0.771 | 0.329 |
|  | Daisy | 17.703 | 16.822 | 6.919 | 6.823 | 5.955 | 5.099 | 0.915 | 1.670 | 4.636 | 3.946 | 1.878 | 1.282 |
|  | Desiree | 23.007 | 19.216 | 7.541 | 7.045 | 4.730 | 4.340 | 1.167 | 2.295 | 3.883 | 2.268 | 1.327 | 0.818 |
|  | Fontane | 12.892 | 10.832 | 3.348 | 3.427 | 11.162 | 7.289 | 0.619 | 1.166 | 1.472 | 1.005 | 0.536 | 0.676 |
|  | Innovator | 8.202 | 7.888 | 5.160 | 5.048 | 7.130 | 5.721 | 1.040 | 2.078 | 2.245 | 1.203 | 1.221 | 0.772 |
|  | King Edward | 19.158 | 16.873 | 7.541 | 8.368 | 17.934 | 6.782 | 1.266 | 3.204 | 3.780 | 2.114 | 1.256 | 0.880 |
|  | Lady Blanca | 19.254 | 15.206 | 9.781 | 8.080 | 8.486 | 5.967 | 1.353 | 2.129 | 1.850 | 1.125 | 1.411 | 0.590 |
|  | Lady Olympia | 18.648 | 19.025 | 7.663 | 9.351 | 8.276 | 7.975 | 1.806 | 3.461 | 2.545 | 1.662 | 1.440 | 0.974 |
|  | Maris Piper | 13.690 | 12.586 | 6.155 | 4.778 | 4.397 | 4.432 | 1.127 | 1.298 | 3.606 | 2.487 | 1.709 | 1.176 |
|  | Markies | 11.618 | 8.895 | 2.550 | 2.268 | 7.663 | 5.889 | 0.619 | 1.414 | 2.957 | 2.452 | 0.768 | 0.634 |
|  | Pentland Dell | 13.841 | 12.549 | 6.124 | 4.305 | 11.503 | 6.823 | 1.450 | 2.540 | 3.066 | 1.229 | 1.012 | 0.498 |
|  | Ramos | 18.987 | 14.078 | 7.296 | 3.814 | 21.005 | 13.179 | 1.036 | 1.767 | 4.186 | 2.957 | 2.037 | 1.165 |
|  | Russet Burbank | 19.643 | 15.297 | 7.983 | 6.204 | 8.623 | 6.248 | 0.877 | 1.592 | 3.727 | 1.848 | 1.600 | 0.513 |
|  | Umatilla Russet | 17.827 | 14.580 | 8.293 | 4.576 | 14.107 | 13.731 | 1.181 | 1.941 | 3.382 | 1.199 | 1.296 | 0.529 |

Table S2. Means tables for variables with variety nested in type by site (Doncaster (Donc) and Woburn (Wob)) interaction effect (*n* = 6)

a. Log_e_ scale unless indicated otherwise

| Type | Variety | Variable and Site | | | | | | | | | | | | | | | |
| --- | --- | --- | --- | --- | --- | --- | --- | --- | --- | --- | --- | --- | --- | --- | --- | --- | --- |
|  |  | Sucrose* | | Alanine | | Glycine | | AABA | | Leucine | | Isoleucine | | Threonine | | Serine | |
|  |  | Donc | Wob | Donc | Wob | Donc | Wob | Donc | Wob | Donc | Wob | Donc | Wob | Donc | Wob | Donc | Wob |
| Boil | Harmony | 3.21 | 3.95 | 0.953 | 0.265 | 0.184 | -0.311 | -2.087 | -3.541 | 1.020 | 0.165 | 1.384 | 0.543 | 1.831 | 0.336 | 1.650 | 0.780 |
| Crisp | Hermes | 18.60 | 27.26 | 1.360 | 1.537 | 0.186 | -0.455 | -1.614 | -2.899 | 0.574 | -0.197 | 1.088 | 0.163 | 1.667 | 0.176 | 1.605 | 0.881 |
|  | Lady Claire | 11.32 | 13.54 | 1.646 | 1.217 | 0.435 | -0.915 | -2.102 | -4.664 | 0.473 | -0.676 | 1.033 | -0.281 | 1.094 | -0.341 | 1.676 | 0.417 |
|  | Lady Rosetta | 20.32 | 20.65 | 1.013 | 0.772 | -0.230 | -0.725 | -2.751 | -4.175 | 0.191 | -0.290 | 0.737 | 0.099 | 0.822 | -0.131 | 1.159 | 0.669 |
|  | Saturna | 17.67 | 16.23 | 0.549 | 0.877 | -0.555 | -1.000 | -2.083 | -3.443 | 0.663 | -0.235 | 0.945 | 0.102 | 0.762 | -0.572 | 0.885 | 0.293 |
|  | Verdi | 14.54 | 18.57 | 0.538 | 0.391 | -0.772 | -1.062 | -2.980 | -3.870 | -0.096 | -0.984 | 0.447 | -0.325 | 0.432 | -0.962 | 0.815 | 0.056 |
| French fry | Challenger | 13.66 | 19.05 | 1.409 | 1.211 | 0.192 | -0.336 | -1.789 | -2.898 | -0.093 | -0.762 | 0.610 | -0.325 | 1.460 | -0.048 | 1.614 | 0.634 |
|  | Daisy | 15.16 | 17.77 | 1.422 | 1.030 | 0.529 | -0.027 | -1.696 | -2.599 | 0.733 | 0.182 | 1.222 | 0.554 | 1.347 | 0.234 | 2.100 | 1.123 |
|  | Desiree | 17.46 | 21.84 | 1.315 | 1.007 | 0.472 | -0.029 | -1.705 | -2.499 | 1.050 | 0.037 | 1.938 | 0.828 | 1.870 | 0.558 | 1.783 | 0.998 |
|  | Fontane | 13.44 | 18.56 | 0.985 | 0.549 | 0.402 | -0.369 | -2.087 | -3.616 | 0.294 | -0.617 | 0.996 | -0.207 | 1.195 | -0.215 | 1.586 | 0.441 |
|  | Innovator | 16.43 | 18.44 | 1.094 | 1.021 | 0.254 | -0.208 | -1.681 | -2.644 | -0.016 | -0.256 | 0.549 | 0.361 | 1.495 | 0.205 | 1.592 | 1.065 |
|  | King Edward | 9.05 | 11.44 | 2.371 | 1.614 | 0.734 | 0.304 | -1.493 | -2.892 | 1.146 | 0.370 | 1.662 | 0.752 | 1.743 | 0.683 | 2.468 | 1.566 |
|  | Lady Blanca | 10.08 | 16.94 | 1.531 | 1.424 | 0.536 | 0.153 | -1.021 | -1.954 | 0.540 | 0.052 | 1.170 | 0.796 | 1.934 | 0.757 | 1.928 | 1.316 |
|  | Lady Olympia | 14.30 | 30.21 | 1.720 | 1.589 | 0.500 | -0.098 | -1.043 | -2.221 | 1.227 | 0.596 | 1.592 | 0.891 | 1.310 | 0.146 | 1.809 | 1.065 |
|  | Maris Piper | 11.90 | 14.87 | 1.115 | 0.823 | 0.590 | -0.143 | -1.386 | -3.022 | 0.726 | 0.115 | 1.457 | 0.505 | 1.308 | 0.403 | 1.867 | 1.120 |
|  | Markies | 13.62 | 15.61 | 1.233 | 0.666 | 0.424 | -0.786 | -2.271 | -3.606 | 0.022 | -0.904 | 0.765 | -0.533 | 1.315 | -0.154 | 1.594 | 0.466 |
|  | Pentland Dell | 17.27 | 18.26 | 1.534 | 1.126 | 0.510 | -0.082 | -1.436 | -2.767 | 0.683 | -0.291 | 1.301 | 0.238 | 1.301 | 0.380 | 1.947 | 1.179 |
|  | Ramos | 12.17 | 17.96 | 1.494 | 1.502 | 0.718 | 0.211 | -1.349 | -2.693 | 0.139 | -0.516 | 0.999 | -0.145 | 1.454 | 0.320 | 1.954 | 1.034 |
|  | Russet Burbank | 14.31 | 17.18 | 1.590 | 1.587 | 0.584 | 0.018 | -0.937 | -1.972 | 0.436 | -0.011 | 1.292 | 0.538 | 1.828 | 0.353 | 2.031 | 1.331 |
|  | Umatilla Russet | 16.94 | 15.21 | 1.809 | 1.016 | 0.604 | -0.138 | -1.114 | -2.345 | 0.798 | -0.026 | 1.419 | 0.472 | 1.389 | 0.230 | 1.938 | 1.039 |
| Within Site SED (df) | | 1.578 (76) | | 0.1920 (76) | | 0.1176 (76) | | 0.2116 (76) | | 0.0940 (76) | | 0.1021 (76) | | 0.1261 (76) | | 0.1355 (76) | |
| LSD (5%) | | 3.143 | | 0.3824 | | 0.2514 | | 0.4214 | | 0.1872 | | 0.2362 | | 0.2511 | | 0.2699 | |
| Other comparisons SED (df) | | 1.560 (79) | | 0.1920 (80) | | 0.1257 (61) | | 0.2105 (79) | | 0.1109 (31) | | 0.1168 (39) | | 0.1315 (71) | | 0.1404 (73) | |
| LSD (5%) | | 3.106 | | 0.3820 | | 0.2342 | | 0.4188 | | 0.2260 | | 0.2362 | | 0.2622 | | 0.2799 | |

*Note: data on raw scale.

Continued:

| Type | Variety | Variable and Site | | | | | | | | | | | | | | | | | |
| --- | --- | --- | --- | --- | --- | --- | --- | --- | --- | --- | --- | --- | --- | --- | --- | --- | --- | --- | --- |
|  |  | Asparagine | | Phenylalanine | | Glutamine | | Ornithine | | Lysine | | Tyrosine | | Tryptophan | | Total | | Asn/Total | |
|  |  | Donc | Wob | Donc | Wob | Donc | Wob | Donc | Wob | Donc | Wob | Donc | Wob | Donc | Wob | Donc | Wob | Donc | Wob |
| Boil | Harmony | 4.619 | 2.831 | 1.303 | 0.483 | 3.783 | 1.685 | -1.594 | -2.997 | 0.796 | 0.427 | 0.312 | -0.094 | 0.159 | -1.205 | 5.567 | 4.487 | -0.948 | -1.655 |
| Crisp | Hermes | 5.033 | 3.240 | 1.215 | 0.305 | 3.985 | 1.940 | -1.870 | -3.348 | 1.267 | 0.705 | 0.804 | 0.587 | -0.105 | -1.235 | 5.775 | 4.588 | -0.742 | -1.348 |
|  | Lady Claire | 4.519 | 2.280 | 1.412 | -0.103 | 4.186 | 1.167 | -1.622 | -4.166 | 0.748 | 0.106 | 0.509 | -0.049 | -0.036 | -1.260 | 5.626 | 4.209 | -1.107 | -1.929 |
|  | Lady Rosetta | 4.519 | 2.537 | 1.259 | 0.523 | 3.258 | 1.415 | -1.010 | -1.706 | 0.653 | 1.002 | 0.112 | -0.099 | -0.606 | -1.319 | 5.342 | 4.308 | -0.823 | -1.772 |
|  | Saturna | 4.981 | 2.617 | 1.069 | -0.047 | 3.287 | 1.140 | -1.392 | -3.146 | 0.646 | 0.714 | 0.191 | 0.202 | -0.421 | -1.103 | 5.602 | 4.174 | -0.621 | -1.557 |
|  | Verdi | 4.661 | 2.015 | 0.784 | -0.220 | 3.004 | 0.934 | -2.184 | -4.264 | -0.270 | 0.115 | -0.401 | -0.684 | -0.308 | -1.634 | 5.313 | 3.920 | -0.652 | -1.906 |
| French fry | Challenger | 4.399 | 2.517 | 1.191 | 0.146 | 4.023 | 1.768 | -1.927 | -3.193 | 0.650 | 0.071 | -0.240 | -0.090 | -0.904 | -2.058 | 5.495 | 4.297 | -1.096 | -1.781 |
|  | Daisy | 4.144 | 2.543 | 1.305 | 0.555 | 3.952 | 1.939 | -0.712 | -1.765 | 1.341 | 1.294 | 0.424 | 0.275 | -0.284 | -1.237 | 5.528 | 4.495 | -1.384 | -1.952 |
|  | Desiree | 5.034 | 3.312 | 1.967 | 0.717 | 4.149 | 2.100 | -1.500 | -2.459 | 1.455 | 1.196 | 1.769 | 1.175 | 0.356 | -0.744 | 5.914 | 4.752 | -0.880 | -1.441 |
|  | Fontane | 4.630 | 2.334 | 0.987 | -0.096 | 3.876 | 1.566 | -1.559 | -2.355 | 0.565 | 0.586 | -0.590 | -0.358 | -1.056 | -1.294 | 5.599 | 4.264 | -0.969 | -1.930 |
|  | Innovator | 4.965 | 2.882 | 0.785 | 0.387 | 4.411 | 2.059 | -1.256 | -1.926 | -0.211 | 1.004 | -0.524 | 0.506 | -2.039 | -0.978 | 5.793 | 4.491 | -0.828 | -1.610 |
|  | King Edward | 4.528 | 2.660 | 1.947 | 0.907 | 4.577 | 2.718 | -1.491 | -2.541 | 1.249 | 1.129 | 1.196 | 1.001 | 0.300 | -0.398 | 5.875 | 4.684 | -1.347 | -2.024 |
|  | Lady Blanca | 5.092 | 3.589 | 1.425 | 0.809 | 4.666 | 2.477 | -2.123 | -2.815 | 0.979 | 0.965 | 0.256 | 0.369 | -0.125 | -0.653 | 5.992 | 4.808 | -0.901 | -1.219 |
|  | Lady Olympia | 4.744 | 3.000 | 1.596 | 0.681 | 4.040 | 1.863 | -1.016 | -2.081 | 1.178 | 1.003 | 0.555 | 0.457 | 0.002 | -1.147 | 5.721 | 4.569 | -0.977 | -1.569 |
|  | Maris Piper | 4.395 | 2.966 | 1.696 | 0.490 | 3.889 | 2.049 | -1.003 | -2.071 | 1.166 | 1.224 | 0.681 | 0.377 | -0.209 | -1.071 | 5.536 | 4.555 | -1.141 | -1.589 |
|  | Markies | 4.823 | 2.917 | 0.690 | -0.418 | 3.888 | 1.645 | -1.466 | -3.679 | 0.282 | 0.231 | -0.576 | -0.918 | -0.429 | -2.132 | 5.641 | 4.322 | -0.818 | -1.405 |
|  | Pentland Dell | 4.693 | 3.104 | 1.760 | 0.783 | 3.418 | 1.994 | -2.177 | -2.730 | 1.148 | 1.274 | 0.753 | 0.401 | -0.577 | -1.526 | 5.635 | 4.640 | -0.943 | -1.536 |
|  | Ramos | 4.899 | 3.322 | 1.164 | 0.053 | 4.349 | 2.565 | -1.129 | -2.765 | 1.027 | 0.947 | -0.113 | -0.223 | -0.798 | -1.272 | 5.870 | 4.670 | -0.972 | -1.348 |
|  | Russet Burbank | 4.851 | 3.502 | 1.518 | 0.610 | 4.152 | 2.099 | -1.546 | -2.543 | 1.119 | 1.069 | 0.859 | 0.951 | -0.152 | -0.645 | 5.807 | 4.793 | -0.955 | -1.291 |
|  | Umatilla Russet | 4.776 | 3.138 | 1.509 | 0.396 | 4.176 | 2.350 | -1.256 | -1.620 | 0.807 | 1.107 | 0.111 | 0.331 | -0.081 | -0.932 | 5.797 | 4.663 | -1.021 | -1.525 |
| Within Site SED (df) | | 0.1397 (76) | | 0.1247 (76) | | 0.2043 (76) | | 0.3925 (76) | | 0.2983 (76) | | 0.2113 (76) | | 0.2324 (76) | | 0.0948 (76) | | 0.0806 (76) | |
| LSD (5%) | | 0.2783 | | 0.2484 | | 0.4070 | | 0.7818 | | 0.5940 | | 0.4209 | | 0.4628 | | 0.1889 | | 0.1606 | |
| Other comparisons SED (df) | | 0.1501 (59) | | 0.1297 (72) | | 0.2096 (76) | | 0.4045 (75) | | 0.2955 (79) | | 0.2177 (75) | | 0.2566 (49) | | 0.0981 (73) | | 0.0871 (57) | |
| LSD (5%) | | 0.3003 | | 0.2586 | | 0.4175 | | 0.8057 | | 0.5880 | | 0.4336 | | 0.5145 | | 0.0196 | | 0.1743 | |

b. Back-transformed means:

| Type | Variety | Variable and Site | | | | | | | | | | | | | |
| --- | --- | --- | --- | --- | --- | --- | --- | --- | --- | --- | --- | --- | --- | --- | --- |
|  |  | Alanine | | Glycine | | AABA | | Leucine | | Isoleucine | | Threonine | | Serine | |
|  |  | Donc | Wob | Donc | Wob | Donc | Wob | Donc | Wob | Donc | Wob | Donc | Wob | Donc | Wob |
| Boil | Harmony | 2.588 | 1.298 | 1.197 | 0.728 | 0.119 | 0.024 | 2.768 | 1.174 | 3.986 | 1.716 | 6.235 | 1.394 | 5.202 | 2.176 |
| Crisp | Hermes | 3.891 | 4.646 | 1.199 | 0.629 | 0.194 | 0.050 | 1.770 | 0.816 | 2.963 | 1.172 | 5.291 | 1.187 | 4.973 | 2.408 |
|  | Lady Claire | 5.181 | 3.372 | 1.540 | 0.396 | 0.117 | 0.004 | 1.600 | 0.504 | 2.804 | 0.750 | 2.981 | 0.706 | 5.339 | 1.512 |
|  | Lady Rosetta | 2.749 | 2.159 | 0.790 | 0.479 | 0.059 | 0.010 | 1.205 | 0.743 | 2.085 | 1.099 | 2.270 | 0.872 | 3.182 | 1.947 |
|  | Saturna | 1.727 | 2.399 | 0.569 | 0.363 | 0.120 | 0.027 | 1.936 | 0.786 | 2.568 | 1.102 | 2.138 | 0.559 | 2.418 | 1.335 |
|  | Verdi | 1.708 | 1.473 | 0.457 | 0.341 | 0.046 | 0.016 | 0.903 | 0.369 | 1.559 | 0.718 | 1.535 | 0.377 | 2.254 | 1.053 |
| French fry | Challenger | 4.087 | 3.352 | 1.207 | 0.710 | 0.162 | 0.050 | 0.906 | 0.462 | 1.835 | 0.718 | 4.301 | 0.948 | 5.018 | 1.880 |
|  | Daisy | 4.140 | 2.796 | 1.692 | 0.968 | 0.178 | 0.069 | 2.076 | 1.195 | 3.389 | 1.735 | 3.841 | 1.259 | 8.161 | 3.069 |
|  | Desiree | 3.720 | 2.732 | 1.598 | 0.966 | 0.177 | 0.077 | 2.853 | 1.033 | 6.940 | 2.284 | 6.483 | 1.742 | 5.943 | 2.708 |
|  | Fontane | 2.673 | 1.727 | 1.490 | 0.686 | 0.119 | 0.022 | 1.337 | 0.535 | 2.702 | 0.808 | 3.299 | 0.802 | 4.879 | 1.549 |
|  | Innovator | 2.981 | 2.771 | 1.284 | 0.807 | 0.181 | 0.066 | 0.979 | 0.769 | 1.727 | 1.430 | 4.454 | 1.223 | 4.909 | 2.896 |
|  | King Edward | 10.703 | 5.018 | 2.078 | 1.350 | 0.220 | 0.050 | 3.141 | 1.443 | 5.265 | 2.116 | 5.709 | 1.975 | 11.794 | 4.782 |
|  | Lady Blanca | 4.618 | 4.149 | 1.704 | 1.160 | 0.355 | 0.137 | 1.711 | 1.048 | 3.217 | 2.212 | 6.912 | 2.127 | 6.871 | 3.723 |
|  | Lady Olympia | 5.580 | 4.894 | 1.644 | 0.902 | 0.347 | 0.104 | 3.406 | 1.810 | 4.909 | 2.433 | 3.701 | 1.152 | 6.099 | 2.896 |
|  | Maris Piper | 3.045 | 2.272 | 1.799 | 0.862 | 0.245 | 0.044 | 2.062 | 1.117 | 4.288 | 1.652 | 3.694 | 1.491 | 6.464 | 3.060 |
|  | Markies | 3.427 | 1.941 | 1.523 | 0.451 | 0.098 | 0.022 | 1.017 | 0.400 | 2.144 | 0.582 | 3.720 | 0.852 | 4.918 | 1.589 |
|  | Pentland Dell | 4.632 | 3.078 | 1.660 | 0.916 | 0.233 | 0.058 | 1.975 | 0.743 | 3.668 | 1.264 | 3.668 | 1.457 | 7.003 | 3.246 |
|  | Ramos | 4.450 | 4.486 | 2.045 | 1.230 | 0.254 | 0.063 | 1.144 | 0.592 | 2.711 | 0.860 | 4.275 | 1.372 | 7.052 | 2.807 |
|  | Russet Burbank | 4.899 | 4.884 | 1.788 | 1.013 | 0.387 | 0.134 | 1.542 | 0.984 | 3.635 | 1.708 | 6.216 | 1.418 | 7.617 | 3.780 |
|  | Umatilla Russet | 6.099 | 2.757 | 1.824 | 0.866 | 0.323 | 0.091 | 2.216 | 0.969 | 4.128 | 1.598 | 4.006 | 1.254 | 6.940 | 2.821 |

Continued:

| Type | Variety | Variable and Site | | | | | | | | | | | | | | | | | |
| --- | --- | --- | --- | --- | --- | --- | --- | --- | --- | --- | --- | --- | --- | --- | --- | --- | --- | --- | --- |
|  |  | Asparagine | | Phenylalanine | | Glutamice | | Ornithine | | Lysine | | Tyrosine | | Tryptophan | | Total | | Asn/Total | |
|  |  | Donc | Wob | Donc | Wob | Donc | Wob | Donc | Wob | Donc | Wob | Donc | Wob | Donc | Wob | Donc | Wob | Donc | Wob |
| Boil | Harmony | 101.388 | 16.957 | 3.675 | 1.616 | 43.943 | 5.387 | 0.198 | 0.045 | 2.212 | 1.528 | 1.361 | 0.905 | 1.167 | 0.295 | 261.643 | 88.849 | 0.383 | 0.186 |
| Crisp | Hermes | 153.388 | 25.529 | 3.365 | 1.352 | 53.780 | 6.954 | 0.149 | 0.030 | 3.545 | 2.019 | 2.229 | 1.794 | 0.895 | 0.286 | 322.139 | 98.293 | 0.471 | 0.255 |
|  | Lady Claire | 91.739 | 9.772 | 4.099 | 0.897 | 65.754 | 3.207 | 0.193 | 0.011 | 2.108 | 1.107 | 1.659 | 0.947 | 0.960 | 0.279 | 277.545 | 67.284 | 0.326 | 0.140 |
|  | Lady Rosetta | 91.739 | 12.637 | 3.517 | 1.682 | 25.992 | 4.111 | 0.359 | 0.177 | 1.916 | 2.719 | 1.114 | 0.901 | 0.541 | 0.262 | 208.925 | 74.287 | 0.434 | 0.165 |
|  | Saturna | 145.615 | 13.690 | 2.907 | 0.949 | 26.757 | 3.122 | 0.244 | 0.038 | 1.903 | 2.037 | 1.205 | 1.219 | 0.651 | 0.327 | 270.963 | 64.970 | 0.532 | 0.206 |
|  | Verdi | 105.737 | 7.496 | 2.185 | 0.798 | 20.161 | 2.540 | 0.108 | 0.009 | 0.758 | 1.117 | 0.665 | 0.500 | 0.730 | 0.190 | 202.953 | 50.395 | 0.516 | 0.144 |
| French fry | Challenger | 81.364 | 12.386 | 3.285 | 1.152 | 55.863 | 5.854 | 0.141 | 0.036 | 1.911 | 1.069 | 0.782 | 0.909 | 0.400 | 0.123 | 243.467 | 73.474 | 0.329 | 0.163 |
|  | Daisy | 63.050 | 12.713 | 3.683 | 1.737 | 52.034 | 6.947 | 0.486 | 0.166 | 3.818 | 3.642 | 1.523 | 1.312 | 0.748 | 0.285 | 251.635 | 89.563 | 0.246 | 0.137 |
|  | Desiree | 153.541 | 27.435 | 7.144 | 2.043 | 63.366 | 8.161 | 0.218 | 0.081 | 4.279 | 3.302 | 5.860 | 3.233 | 1.423 | 0.470 | 370.179 | 115.811 | 0.410 | 0.232 |
|  | Fontane | 102.509 | 10.314 | 2.678 | 0.903 | 48.226 | 4.782 | 0.205 | 0.090 | 1.754 | 1.792 | 0.549 | 0.694 | 0.343 | 0.269 | 270.151 | 71.089 | 0.374 | 0.140 |
|  | Innovator | 143.304 | 17.845 | 2.187 | 1.468 | 82.347 | 7.833 | 0.280 | 0.141 | 0.805 | 2.724 | 0.587 | 1.654 | 0.125 | 0.371 | 327.991 | 89.206 | 0.432 | 0.195 |
|  | King Edward | 92.568 | 14.291 | 7.003 | 2.472 | 97.217 | 15.145 | 0.220 | 0.074 | 3.482 | 3.088 | 3.302 | 2.716 | 1.345 | 0.667 | 356.020 | 108.197 | 0.255 | 0.127 |
|  | Lady Blanca | 162.710 | 36.193 | 4.153 | 2.241 | 106.267 | 11.900 | 0.115 | 0.055 | 2.657 | 2.620 | 1.287 | 1.441 | 0.877 | 0.515 | 400.209 | 122.481 | 0.401 | 0.291 |
|  | Lady Olympia | 114.888 | 20.081 | 4.928 | 1.971 | 56.821 | 6.438 | 0.357 | 0.120 | 3.243 | 2.721 | 1.737 | 1.574 | 0.997 | 0.313 | 305.205 | 96.443 | 0.371 | 0.203 |
|  | Maris Piper | 81.040 | 19.409 | 5.447 | 1.627 | 48.857 | 7.755 | 0.362 | 0.121 | 3.204 | 3.396 | 1.971 | 1.453 | 0.806 | 0.338 | 253.656 | 95.102 | 0.314 | 0.199 |
|  | Markies | 124.333 | 18.481 | 1.989 | 0.653 | 48.808 | 5.176 | 0.226 | 0.020 | 1.321 | 1.255 | 0.557 | 0.394 | 0.646 | 0.114 | 281.739 | 75.334 | 0.436 | 0.240 |
|  | Pentland Dell | 109.175 | 22.282 | 5.807 | 2.183 | 30.503 | 7.340 | 0.108 | 0.060 | 3.147 | 3.570 | 2.118 | 1.488 | 0.557 | 0.212 | 280.054 | 103.539 | 0.384 | 0.210 |
|  | Ramos | 134.151 | 27.711 | 3.198 | 1.049 | 77.396 | 12.996 | 0.318 | 0.058 | 2.788 | 2.573 | 0.888 | 0.795 | 0.445 | 0.275 | 354.244 | 106.693 | 0.373 | 0.255 |
|  | Russet Burbank | 127.863 | 33.177 | 4.558 | 1.835 | 63.556 | 8.153 | 0.208 | 0.074 | 3.057 | 2.907 | 2.356 | 2.583 | 0.854 | 0.520 | 332.615 | 120.658 | 0.380 | 0.270 |
|  | Umatilla Russet | 118.624 | 23.053 | 4.517 | 1.481 | 65.100 | 10.481 | 0.280 | 0.193 | 2.236 | 3.020 | 1.112 | 1.387 | 0.917 | 0.389 | 329.305 | 105.948 | 0.355 | 0.213 |

Table S3. Means table for variables with a site by type by storage interaction, on the Log_e_ scale with back-transformed means in brackets (*n* variable as indicated)

| Site | Type  (replication, *n*) | Variable and Storage | | | | | |
| --- | --- | --- | --- | --- | --- | --- | --- |
|  |  | AABA | | Asparagine | | Asparagine/Total | |
|  |  | 2 months storage | 6 months storage | 2 months storage | 6 months storage | 2 months storage | 6 months storage |
| Doncaster | Boil (3) | -2.008 (0.129) | -2.165 (0.110) | 4.674 (107.120) | 4.565 (96.058) | -0.846 (0.424) | -1.050 (0.345) |
|  | Crisp (15) | -1.944 (0.138) | -2.668 (0.064) | 4.812 (122.972) | 4.673 (107.013) | -0.752 (0.466) | -0.827 (0.432) |
|  | Fr. fry (42) | -1.250 (0.282) | -1.751 (0.169) | 4.841 (126.591) | 4.583 (97.802) | -0.944 (0.384) | -1.089 (0.332) |
| Woburn | Boil (3) | -2.856 (0.052) | -4.225 (0.010) | 3.078 (21.710) | 2.585 (13.258) | -1.491 (0.220) | -1.820 (0.157) |
|  | Crisp (15) | -2.875 (0.051) | -4.746 (0.004) | 2.785 (16.195) | 2.290 (9.870) | -1.614 (0.194) | -1.791 (0.162) |
|  | Fr. fry (42) | -2.200 (0.106) | -3.190 (0.036) | 3.114 (22.506) | 2.855 (17.369) | -1.578 (0.201) | -1.596 (0.198) |
| Within Site and Storage SEDs (df), LSDs  Boil vs. Crisp (3 vs. 15 rep.)  Boil vs. Fr. fry (3 vs. 42 rep.)  Crisp vs. Fr. fry (15 vs. 42 rep.) | | 0.2268 (155), 0.4482  0.2143 (155), 0.4234  0.1079 (155), 0.2132 | | 0.1324 (137), 0.2618  0.1251 (137), 0.2474  0.0630 (137), 0.1246 | | 0.0918 (142), 0.1815  0.0738 (142), 0.1459  0.0371 (142), 0.0733 | |
| Within Site and Type SED (df), LSD  Boil (3 vs. 3 rep.)  Crisp (15 vs. 15 rep.)  Fr. fry (42 vs. 42 rep.) | | 0.2863 (80), 0.5698  0.2096 (80), 0.4170  0.0765 (80), 0.1523 | | 0.1392 (80), 0.2771  0.1019 (80), 0.2028  0.0372 (80), 0.0741 | | 0.0856 (80), 0.1703  0.0626 (80), 0.1246  0.0229 (80), 0.0455 | |
| Other comparisons  Boil (3 vs. 3 rep.)  Boil vs. Crisp (3 vs 15 rep.)  Boil vs. Fr. fry (3 vs 42 rep.)  Boil (15 vs. 15 rep.)  Boil vs. Fr. fry (15 vs. 42 rep.)  Fr. fry (42 vs. 42 rep.) | | 0.2920 (159), 0.5767  0.2262 (158), 0.4467  0.2132 (158), 0.4211  0.1258 (102), 0.2495  0.1037 (70), 0.2068  0.0752 (35), 0.1526 | | 0.1795 (107), 0.3559  0.1390 (90), 0.2762  0.1366 (63), 0.2730  0.1240 (40), 0.2505  0.1021 (22), 0.2118  0.0741 (6), 0.1714 | | 0.1060 (108), 0.2102  0.0821 (94), 0.1631  0.0808 (63), 0.1614  0.0494 (35), 0.1003  0.0461 (24), 0.0950  0.0425 (6), 0.1018 | |

Table S4. Means table for **v**ariables with variety nested in type by storage (2 or 6 months) interaction (*n* = 6)

a. Log_e_ scale

| Type | Variety | Variable and Storage | | | | | | | | | | | | | | | |
| --- | --- | --- | --- | --- | --- | --- | --- | --- | --- | --- | --- | --- | --- | --- | --- | --- | --- |
|  |  | Sucrose* | | AABA | | Leucine | | Isoleucine | | Phenylalanine | | Tyrosine | | Tryptophan | | Asn/Total | |
|  |  | 2 | 6 | 2 | 6 | 2. | 6 | 2. | 6 | 2. | 6 | 2. | 6 | 2. | 6 | 2. | 6 |
| Boil | Harmony | 4.23 | 2.93 | -2.432 | -3.195 | 0.438 | 0.746 | 0.906 | 1.021 | 1.037 | 0.749 | 0.113 | 0.105 | -0.586 | -0.460 | -1.168 | -1.435 |
| Crisp | Hermes | 25.15 | 20.71 | -1.762 | -2.752 | 0.197 | 0.181 | 0.737 | 0.515 | 1.019 | 0.502 | 0.763 | 0.628 | -0.594 | -0.745 | -0.969 | -1.121 |
|  | Lady Claire | 15.94 | 8.92 | -2.986 | -3.780 | -0.266 | 0.063 | 0.355 | 0.397 | 0.859 | 0.450 | 0.178 | 0.282 | -0.569 | -0.728 | -1.464 | -1.572 |
|  | Lady Rosetta | 16.83 | 24.14 | -2.717 | -4.208 | -0.135 | 0.036 | 0.463 | 0.373 | 0.867 | 0.914 | -0.105 | 0.118 | -0.728 | -1.197 | -1.237 | -1.358 |
|  | Saturna | 19.41 | 14.49 | -1.945 | -3.581 | 0.155 | 0.274 | 0.545 | 0.502 | 0.701 | 0.322 | 0.172 | 0.221 | -0.769 | -0.754 | -1.074 | -1.105 |
|  | Verdi | 18.08 | 15.03 | -2.638 | -4.213 | -0.664 | -0.415 | 0.015 | 0.107 | 0.459 | 0.106 | -0.563 | -0.522 | -0.911 | -1.031 | -1.169 | -1.389 |
| French fry | Challenger | 16.60 | 16.11 | -2.036 | -2.650 | -0.526 | -0.328 | 0.252 | 0.034 | 0.945 | 0.391 | -0.193 | -0.136 | -1.334 | -1.628 | -1.309 | -1.568 |
|  | Daisy | 18.63 | 14.31 | -1.785 | -2.510 | 0.385 | 0.530 | 0.912 | 0.864 | 1.011 | 0.850 | 0.209 | 0.490 | -0.744 | -0.777 | -1.579 | -1.758 |
|  | Desiree | 19.34 | 19.97 | -1.643 | -2.561 | 0.568 | 0.519 | 1.457 | 1.308 | 1.698 | 0.986 | 1.656 | 1.287 | -0.074 | -0.314 | -1.158 | -1.163 |
|  | Fontane | 16.08 | 15.92 | -2.240 | -3.464 | -0.236 | -0.087 | 0.471 | 0.318 | 0.537 | 0.354 | -0.575 | -0.373 | -1.059 | -1.291 | -1.418 | -1.482 |
|  | Innovator | 14.76 | 20.11 | -1.994 | -2.331 | -0.251 | -0.022 | 0.400 | 0.510 | 0.773 | 0.399 | -0.191 | 0.173 | -2.133 | -0.883 | -1.177 | -1.260 |
|  | King Edward | 12.57 | 7.92 | -1.664 | -2.721 | 0.714 | 0.802 | 1.230 | 1.184 | 1.546 | 1.308 | 0.982 | 1.216 | 0.016 | -0.113 | -1.570 | -1.800 |
|  | Lady Blanca | 12.65 | 14.38 | -1.222 | -1.753 | 0.260 | 0.332 | 1.080 | 0.887 | 1.404 | 0.830 | 0.341 | 0.284 | -0.298 | -0.480 | -1.066 | -1.054 |
|  | Lady Olympia | 22.50 | 22.01 | -1.468 | -1.796 | 0.778 | 1.045 | 1.163 | 1.320 | 1.146 | 1.131 | 0.324 | 0.689 | -0.683 | -0.462 | -1.188 | -1.358 |
|  | Maris Piper | 18.81 | 7.95 | -1.623 | -2.785 | 0.504 | 0.337 | 1.098 | 0.863 | 1.332 | 0.853 | 0.579 | 0.480 | -0.479 | -0.801 | -1.361 | -1.369 |
|  | Markies | 16.21 | 13.02 | -2.649 | -3.228 | -0.409 | -0.474 | 0.282 | -0.050 | 0.448 | -0.176 | -0.513 | -0.981 | -0.838 | -1.723 | -1.073 | -1.150 |
|  | Pentland Dell | 15.46 | 20.07 | -1.615 | -2.588 | 0.227 | 0.165 | 0.902 | 0.637 | 1.455 | 1.088 | 0.713 | 0.440 | -0.867 | -1.236 | -1.180 | -1.298 |
|  | Ramos | 15.86 | 14.27 | -1.595 | -2.447 | -0.085 | -0.291 | 0.708 | 0.147 | 0.925 | 0.291 | 0.058 | -0.393 | -0.844 | -1.226 | -1.154 | -1.166 |
|  | Russet Burbank | 18.39 | 13.10 | -1.175 | -1.734 | 0.279 | 0.145 | 1.040 | 0.790 | 1.374 | 0.754 | 1.074 | 0.737 | -0.239 | -0.558 | -1.136 | -1.110 |
|  | Umatilla Russet | 15.53 | 16.62 | -1.443 | -2.017 | 0.500 | 0.272 | 1.163 | 0.728 | 1.393 | 0.512 | 0.602 | -0.159 | -0.297 | -0.715 | -1.288 | -1.258 |
| Within Type and Variety SED (df) | | 1.712 (80) | | 0.2025 (80) | | 0.0864 (80) | | 0.0808 (80) | | 0.1034 (80) | | 0.1672 (80) | | 0.2222 (80) | | 0.0605 (80) | |
| LSD (5%) | | 3.252 | | 0.4029 | | 0.1718 | | 0.1607 | | 0.2058 | | 0.3327 | | 0.4491 | | 0.1204 | |
| Other comparisons SED (df) | | 1.646 (155) | | 0.2071 (155) | | 0.0903 (154) | | 0.0921 (146) | | 0.1146 (149) | | 0.1905 (146) | | 0.2274 (155) | | 0.0713 (142) | |
| LSD (5%) | | 3.406 | | 0.4090 | | 0.1783 | | 0.1820 | | 0.2264 | | 0.3765 | | 0.4422 | | 0.1409 | |

*Note: data on raw scale.

Back-transformed means:

| Type | Variety | Variable and Storage | | | | | | | | | | | | | |
| --- | --- | --- | --- | --- | --- | --- | --- | --- | --- | --- | --- | --- | --- | --- | --- |
|  |  | AABA | | Leucine | | Isoleucine | | Phenylalanine | | Tyrosine | | Tryptophan | | Asn/Total | |
|  |  | 2 | 6 | 2 | 6 | 2. | 6 | 2. | 6 | 2. | 6 | 2. | 6 | 2. | 6 |
| Boil | Harmony | 0.083 | 0.036 | 1.545 | 2.104 | 2.469 | 2.771 | 2.816 | 2.110 | 1.115 | 1.106 | 0.552 | 0.626 | 0.306 | 0.233 |
| Crisp | Hermes | 0.167 | 0.059 | 1.213 | 1.193 | 2.085 | 1.669 | 2.765 | 1.647 | 2.140 | 1.869 | 0.547 | 0.470 | 0.374 | 0.321 |
|  | Lady Claire | 0.045 | 0.018 | 0.761 | 1.060 | 1.421 | 1.482 | 2.356 | 1.563 | 1.190 | 1.321 | 0.561 | 0.478 | 0.226 | 0.203 |
|  | Lady Rosetta | 0.061 | 0.010 | 0.869 | 1.032 | 1.584 | 1.447 | 2.375 | 2.489 | 0.895 | 1.120 | 0.478 | 0.297 | 0.285 | 0.252 |
|  | Saturna | 0.138 | 0.023 | 1.163 | 1.310 | 1.720 | 1.647 | 2.011 | 1.375 | 1.183 | 1.242 | 0.458 | 0.465 | 0.337 | 0.326 |
|  | Verdi | 0.067 | 0.010 | 0.510 | 0.655 | 1.010 | 1.108 | 1.577 | 1.107 | 0.564 | 0.588 | 0.397 | 0.352 | 0.306 | 0.244 |
| French fry | Challenger | 0.126 | 0.066 | 0.586 | 0.715 | 1.282 | 1.030 | 2.568 | 1.473 | 0.819 | 0.868 | 0.258 | 0.191 | 0.265 | 0.203 |
|  | Daisy | 0.163 | 0.076 | 1.465 | 1.694 | 2.484 | 2.368 | 2.743 | 2.335 | 1.227 | 1.627 | 0.470 | 0.455 | 0.201 | 0.167 |
|  | Desiree | 0.188 | 0.072 | 1.760 | 1.675 | 4.288 | 3.694 | 5.458 | 2.675 | 5.233 | 3.617 | 0.924 | 0.726 | 0.309 | 0.308 |
|  | Fontane | 0.101 | 0.026 | 0.785 | 0.912 | 1.597 | 1.369 | 1.706 | 1.420 | 0.558 | 0.684 | 0.342 | 0.270 | 0.237 | 0.222 |
|  | Innovator | 0.131 | 0.092 | 0.773 | 0.973 | 1.487 | 1.660 | 2.161 | 1.485 | 0.821 | 1.184 | 0.113 | 0.409 | 0.303 | 0.279 |
|  | King Edward | 0.184 | 0.061 | 2.037 | 2.225 | 3.416 | 3.262 | 4.688 | 3.694 | 2.665 | 3.369 | 1.011 | 0.888 | 0.203 | 0.160 |
|  | Lady Blanca | 0.290 | 0.168 | 1.292 | 1.389 | 2.940 | 2.423 | 4.066 | 2.288 | 1.401 | 1.323 | 0.737 | 0.614 | 0.339 | 0.344 |
|  | Lady Olympia | 0.225 | 0.161 | 2.172 | 2.838 | 3.195 | 3.738 | 3.141 | 3.094 | 1.378 | 1.987 | 0.500 | 0.625 | 0.300 | 0.252 |
|  | Maris Piper | 0.192 | 0.057 | 1.650 | 1.396 | 2.993 | 2.365 | 3.784 | 2.342 | 1.779 | 1.611 | 0.614 | 0.444 | 0.251 | 0.249 |
|  | Markies | 0.066 | 0.035 | 0.659 | 0.618 | 1.321 | 0.946 | 1.560 | 0.834 | 0.594 | 0.370 | 0.428 | 0.174 | 0.337 | 0.312 |
|  | Pentland Dell | 0.194 | 0.070 | 1.250 | 1.174 | 2.460 | 1.886 | 4.279 | 2.963 | 2.035 | 1.548 | 0.415 | 0.286 | 0.302 | 0.268 |
|  | Ramos | 0.198 | 0.082 | 0.914 | 0.743 | 2.025 | 1.153 | 2.517 | 1.333 | 1.055 | 0.670 | 0.425 | 0.288 | 0.310 | 0.307 |
|  | Russet Burbank | 0.304 | 0.172 | 1.317 | 1.151 | 2.824 | 2.198 | 3.946 | 2.120 | 2.922 | 2.085 | 0.782 | 0.567 | 0.316 | 0.325 |
|  | Umatilla Russet | 0.231 | 0.128 | 1.644 | 1.308 | 3.195 | 2.066 | 4.022 | 1.664 | 1.821 | 0.848 | 0.738 | 0.484 | 0.271 | 0.279 |

Table S5. Means table for variables with site by storage effect, on the Log_e_ scale unless indicated, with back-transformed means in brackets (*n* = 60)

| Variable | Site and Storage | | | | Within site  SED (df) LSD (5%) | | Other Comparisons  SED (df) LSD (5%) | |
| --- | --- | --- | --- | --- | --- | --- | --- | --- |
|  | Doncaster | | Woburn | |  |  |  |  |
|  | 2 months storage | 6 months storage | 2 months storage | 6 months storage |  |  |  |  |
| Sucrose* | 14.31 | 13.83 | 18.99 | 16.37 | 0.541 (80) | 1.077 | 0.465 (31) | 0.947 |
| Alanine | 1.486 (4.414) | 1.182 (3.256) | 0.733 (2.076) | 1.390 (4.010) | 0.0594 (80) | 0.1181 | 0.0600 (14) | 0.1283 |
| Leucine | 0.518 (1.674) | 0.533 (1.699) | -0.274 (0.755) | -0.150 (0.856) | 0.0273 (80) | 0.0543 | 0.0654 (4) | 0.1703 |
| Isoleucine | 1.157 (3.175) | 1.104 (3.011) | 0.361 (1.430) | 0.142 (1.148) | 0.0255 (80) | 0.0508 | 0.0636 (4) | 0.1664 |
| Threonine | 1.313 (3.712) | 1.443 (4.228) | 0.230 (1.254) | 0.005 (1.000) | 0.0339 (80) | 0.0675 | 0.0527 (6) | 0.1272 |
| Serine | 1.577 (4.835) | 1.823 (6.185) | 0.960 (2.607) | 0.788 (2.194) | 0.0384 (80) | 0.0765 | 0.0549 (6) | 0.1300 |
| Aspartic acid | 3.194 (24.381) | 3.318 (27.600) | 3.134 (22.961) | 2.648 (14.121) | 0.0594 (80) | 0.1182 | 0.0640 (12) | 0.1395 |
| Phenylalanine | 1.618 (5.038) | 1.040 (2.824) | 0.475 (1.603) | 0.222 (1.244) | 0.0327 (80) | 0.0651 | 0.0508 (6) | 0.1227 |
| Glutamine | 3.854 (47.176) | 4.053 (57.565) | 1.893 (6.634) | 1.854 (6.380) | 0.0578 (80) | 0.1151 | 0.0771 (7) | 0.1790 |
| Ornithine | -1.691 (0.179) | -1.292 (0.270) | -2.552 (0.073) | -2.865 (0.052) | 0.1254 (80) | 0.2497 | 0.1584 (8) | 0.3623 |
| Lysine | 0.454 (1.570) | 1.206 (3.3350 | 0.998 (2.708) | 0.618 (1.850) | 0.0816 (80) | 0.1623 | 0.0782 (18) | 0.1643 |
| Histidine | -1.070 (0.338) | 0.222 (1.244) | -0.029 (0.966) | -0.173 (0.836) | 0.1062 (80) | 0.2113 | 0.1196 (10) | 0.2642 |
| Tyrosine | 0.159 (1.167) | 0.450 (1.563) | 0.404 (1.493) | 0.008 (1.003) | 0.0529 (80) | 0.1052 | 0.0797 (6) | 0.1912 |
| Tryptophan | -0.460 (0.626) | -0.271 (0.758) | -0.943 (0.384) | -1.441 (0.232) | 0.0703 (80) | 0.1398 | 0.1304 (5) | 0.3267 |
| Total | 5.717 (303.987) | 5.626 (277.545) | 4.613 (100.781) | 4.356 (77.940) | 0.0229 (80) | 0.0455 | 0.0366 (6) | 0.0891 |

*Note: data on raw scale.

Table S6. Means table for variables with variety nested in type effect, on the Log_e_ scale with back-transformed means in brackets (*n* = 12)

| Type | Variety | Variable | | |
| --- | --- | --- | --- | --- |
|  |  | GABA | Aspartic acid | Histidine |
| Boil | Harmony | 2.572 (13.087) | 3.260 (26.045) | -0.629 (0.528) |
| Crisp | Hermes | 2.589 (13.311) | 3.042 (20.942) | -0.521 (0.589) |
|  | Lady Claire | 2.020 (7.533) | 3.021 (20.507) | -0.342 (0.705) |
|  | Lady Rosetta | 2.346 (10.439) | 2.824 (16.839) | -0.141 (0.863) |
|  | Saturna | 2.073 (7.944) | 2.962 (19.332) | -0.769 (0.458) |
|  | Verdi | 1.605 (4.973) | 2.665 (14.363) | -0.885 (0.408) |
| French fry | Challenger | 2.021 (7.541) | 3.221 (25.048) | -0.690 (0.497) |
|  | Daisy | 2.683 (14.624) | 3.280 (26.571) | 0.032 (1.028) |
|  | Desiree | 2.625 (13.800) | 3.301 (27.135) | 0.150 (1.157) |
|  | Fontane | 2.468 (11.794) | 3.150 (23.331) | -0.348 (0.701) |
|  | Innovator | 2.024 (7.564) | 2.984 (19.762) | -0.627 (0.529) |
|  | King Edward | 2.566 (13.009) | 2.736 (15.420) | 0.169 (1.179) |
|  | Lady Blanca | 2.224 (9.239) | 3.008 (20.242) | 0.061 (1.058) |
|  | Lady Olympia | 1.837 (6.273) | 2.923 (18.592) | 0.066 (1.063) |
|  | Maris Piper | 2.652 (14.177) | 3.047 (21.047) | -0.407 (0.661) |
|  | Markies | 2.004 (7.414) | 2.989 (19.861) | -0.245 (0.778) |
|  | Pentland Dell | 2.387 (10.876) | 3.385 (29.513) | -0.216 (0.801) |
|  | Ramos | 2.426 (11.309) | 3.132 (22.915) | 0.182 (1.195) |
|  | Russet Burbank | 2.857 (17.404) | 3.131 (22.892) | 0.143 (1.149) |
|  | Umatilla Russet | 1.961 (7.101) | 3.407 (30.170) | -0.233 (0.787) |
| SED (df) | | 0.2816 (76) | 0.1447 (76) | 0.2727 (76) |
| LSD (5%) | | 0.5608 | 0.2881 | 0.5432 |

Table S7. Means table for variable (glycine concentration) with a main effect of storage, on the Log_e_ scale with back-transformed means in brackets (*n* = 120)

|  | Glycine | |
| --- | --- | --- |
|  | 2 months storage | 6 months storage |
|  | 0.076 (1.074) | -0.061 (0.941) |
| SED (df), LSD (5%) | 0.0231 (80), 0.0459 | |

Table S8. Variables with site by type interaction effect, on the Log_e_ scale with back-transformed means in brackets (*n* variable as indicated)

| Site | Type (replication, *n*) | GABA |
| --- | --- | --- |
| Doncaster | Boil (6) | 2.990 (19.881) |
|  | Crisp (30) | 2.753 (15.685) |
|  | Fr. fry (84) | 2.654 (14.206) |
| Woburn | Boil (6) | 2.153 (8.606) |
|  | Crisp (30) | 1.500 (4.477) |
|  | Fr. fry (84) | 2.022 (7.548) |
| Within site SEDs (df), LSDs  Boil vs. Fr. fry: 0.2915 (76), 0.5805  Boil vs. Crisp: 0.3084 (76), 0.6143  Crisp vs. Fr. fry: 0.1467 (76), 0.2921 | | |
| Other comparisons SEDs (df), LSDs  6 rep. vs. 6 rep.: 0.4041 (78), 0.8045  6 rep. vs. 30 rep.: 0.3130 (73), 0.6230  6 rep. vs. 84 rep.: 0.2995 (68), 0.5977  30 rep. vs. 84 rep.: 0.1507 (37), 0.3054  84 rep. vs. 84 rep.: 0.1268 (6), 0.3056 | | |
